# *rcm1t*, a Loss-of-Function Allele of *SlRCM1*, Causes Premature Chloroplast Senescence and Is Associated with Enhanced Osmotic Stress Tolerance in Tomato

**DOI:** 10.3390/ijms27188071

**Published:** 2026-09-10

**Authors:** Zhuoyao Qiu, Chunyang Pan, Dongling Lan, Wenzheng Gao, Wen Liu, Qingmei Wang, Can Zhu, Xin Li, Lei Liu, Zejun Huang, Yongchen Du, Junming Li, Wencai Yang, Xiaoxuan Wang

**Affiliations:** 1 State Key Laboratory of Vegetable Biobreeding, Institute of Vegetables and Flowers, Chinese Academy of Agricultural Sciences, Beijing 100081, China; 82101229106@caas.cn (Z.Q.); panchunyang@taas.ac.cn (C.P.); 821012590024@caas.cn (D.L.); gaowenzheng@caas.cn (W.G.); 821012430276@caas.cn (W.L.); 821012550181@caas.cn (Q.W.); zhucan@caas.cn (C.Z.); lixin10@caas.cn (X.L.); liulei02@caas.cn (L.L.); huangzejun@caas.cn (Z.H.); duyongchen@caas.cn (Y.D.); lijunming@caas.cn (J.L.); 2 Department of Vegetable Science, College of Horticulture, China Agricultural University, Beijing 100193, China; 3 State Key Laboratory of Vegetable Biobreeding, Tianjin Academy of Agricultural Sciences, Tianjin 300192, China

**Keywords:** tomato, chloroplast senescence, transcriptome analysis, abscisic acid, osmotic stress resistance

## Abstract

Chloroplasts drive photosynthesis and regulate cellular signaling and phytohormone biosynthesis processes that greatly influence crop yield and quality traits. In this study, we identified a recessive chlorophyll-deficient mutant, *reduced chlorophyll mutant 1t* (*rcm1t*), from an advanced-generation tomato inbred line. Genetic and phenotypic characterization confirmed that chloroplast degradation accelerates rapidly and chlorosis occurs during late leaf development in the *rcm1t* mutant. Subsequently, we fine-mapped *rcm1t* to an approximately 60 kb region on chromosome 8. This interval encompasses *Lutescent* (*L1*)/*SlRCM1*, a well-characterized gene essential for chloroplast biogenesis and developmental maintenance. An allelism test between *rcm1t* and the previously characterized ethyl methanesulfonate (EMS)-induced *rcm1* mutant revealed non-complementation. Moreover, transgenic complementation with the wild-type *SlRCM1* coding sequence rescued the chloroplast premature senescence phenotype in *rcm1t*, providing definitive genetic evidence that *rcm1t* is a loss-of-function allele of *SlRCM1*. Resequencing identified a ~4.8 kb retrotransposon insertion in the first exon of the gene, which disrupts normal transcription and results in a premature stop codon. Quantification of abscisic acid (ABA) revealed significantly higher ABA levels in the *rcm1t* mutant than in the wild type. Protein–protein interaction analyses demonstrate that SlRCM1 interacts with SlNCED2. Osmotic stress assays showed that the *rcm1t* near-isogenic line exhibited enhanced tolerance under osmotic stress. This study successfully mapped the natural allele *rcm1t* of *SlRCM1*, preliminarily explored its potential application in enhancing plant tolerance to osmotic stress, and provided valuable insights for further elucidation of *SlRCM1* function.

## 1. Introduction

Tomato (*Solanum lycopersicum*) is one of the world’s most economically important vegetable crops, and its commercial value is determined by multiple agronomic and postharvest traits, including yield, fruit quality, and shelf life [1,2,3]. Chloroplast development and senescence are particularly important for both yield formation and plant responses to biotic and abiotic stresses [4,5]. Chloroplasts are highly specialized, semi-autonomous organelles unique to plant cells. As the primary sites of photosynthesis, they coordinate light harvesting, photosynthetic electron transport, and carbon fixation. In addition, chloroplasts serve as major metabolic hubs for the biosynthesis of fatty acids, amino acids, tetrapyrroles, carotenoids, and precursors of several phytohormones [6,7].

Consequently, chloroplast dysfunction, defects in thylakoid membrane system assembly, and imbalances in chlorophyll homeostasis not only lead to reduced photosynthetic efficiency and accumulation of reactive oxygen species (ROS), but also significantly disrupt the coordinated balance between vegetative growth and reproductive development, ultimately compromising crop yield and quality. For example, the rice (*Oryza sativa*) chlorophyll-deficient mutant *yellow-green leaf1* (*ygl1*) exhibits delayed chloroplast development, diminished vegetative growth, and significantly reduced chlorophyll and carotenoid accumulation [8]. Similarly, the yellow-leaf mutant (*YX-yl*) of broomcorn millet (*Panicum miliaceum*) displays significantly reduced plant height and markedly lower grain yield than the wild type [9]. In tomato (*S. lycopersicum*), chloroplasts in immature green fruits differ functionally from leaf chloroplasts, which constitute the principal photosynthetic apparatus of the plant [4,10,11,12]. Fruit chloroplasts directly contribute to fruit expansion, ripening progression, and quality trait formation by integrating photosynthetic carbon assimilation with the coordinated biosynthesis and accumulation of starch and secondary metabolites. Therefore, mutations in the key genes involved in chloroplast senescence in tomatoes significantly impair fruit yield and quality [10,11]. The tomato *Lutescent2* (*L2*) gene encodes a chloroplast-targeted M50-family zinc metalloprotease [10]. Loss-of-function mutations in *L2* impair chlorophyll biosynthesis during de-etiolation and enhance chlorophyll catabolism during leaf and fruit senescence, resulting in premature senescence and substantial yield loss [10,11]. Loss-of-function mutations in the *Lutescent1* (*L1*)/*SlRCM1* gene, which encodes a chloroplast-targeted metalloendopeptidase, induce premature chloroplast senescence during fruit ripening and significant reductions in fruit carotenoid and soluble solids contents, as well as decreased pollen viability and an increased frequency of abnormal pollen grains [12].

However, emerging evidence indicates that not all mutations impairing chloroplast function confer deleterious effects. Under specific environmental conditions or genetic backgrounds, certain mutations that disrupt chloroplast development or accelerate chloroplast senescence may confer adaptive or agronomically beneficial traits. In soybean (*Glycine max*), the stay-green G protein (GmG) regulates seed dormancy by physically interacting with NCED3 and PSY proteins, thereby modulating abscisic acid (ABA) biosynthesis [13]. Loss-of-function mutations in *GmG* significantly enhance the germination rate of freshly harvested seeds [13]. Similarly, loss-of-function mutations in its *Arabidopsis thaliana* ortholog, *AtG*, produce comparable phenotypic effects, including reduced seed dormancy and accelerated germination [13,14]. In maize (*Zea mays*), *ZmCAAX* encodes a CAAX amino terminal protease family protein. Loss-of-function mutations in *ZmCAAX* induce premature chloroplast senescence and leaf yellowing [15]. However, disruption of ZmCAAX reduces the degradation of ZmNCED3, a key enzyme in ABA biosynthesis, thereby promoting carotenoid cleavage and ABA accumulation. Ultimately, these effects enhance plant drought tolerance and increase carotenoid content in grains [15].

CaaX proteases are involved in protein prenylation. Prenylation is a post-translational modification in which protein prenyltransferases recognize a C-terminal CaaX motif (C = cysteine, A = aliphatic amino acid, X = any amino acid) and catalyze the covalent attachment of either a farnesyl (C15) or geranylgeranyl (C20) isoprenoid group to the cysteine residue. Following prenylation, CaaX proteases remove the terminal -aaX tripeptide from the modified substrate protein [15,16,17,18]. This essential post-translational modification regulates a wide range of cellular processes by controlling the subcellular localization, protein–protein interactions, and stability of modified target proteins [18]. For example, in *A. thaliana*, the CaaX protease-dependent BALANCE of CHLOROPHYLL METABOLISM (BCM) pathway delays chlorophyll catabolism during leaf senescence by degradation of Stay-Green 1 (SGR1), a central positive regulator of chlorophyll catabolism [19]. Moreover, geranylgeranylation of the petunia calmodulin CaM53 promotes its relocalization from the nucleus to the plasma membrane, a process that is essential for its function in calcium signaling [20,21].

In tomato, loss-of-function mutations in *L1/SlRCM1* have been reported to cause pronounced chlorophyll-deficient phenotypes, with fully expanded leaves exhibiting conspicuous chlorosis [12]. Previous studies further showed that disruption of *SlRCM1* leads to abnormal chloroplast morphology and impaired thylakoid organization in immature fruits, suggesting an important role for *SlRCM1* in chloroplast development [12]. However, it remains unclear whether the chlorotic phenotype associated with *SlRCM1* deficiency primarily arises from defects in early chloroplast development or from the premature degradation of chloroplasts that initially develop normally. Notably, *SlRCM1* is the tomato ortholog of *GmG* and *ZmCAAX*, both of which have been implicated in ABA-related physiological processes [13,15]. However, whether *SlRCM1* is involved in ABA regulation and plant responses to osmotic stress in tomato remains largely unknown, with direct genetic and physiological evidence still lacking.

In this study, we identified *rcm1t*, a naturally occurring loss-of-function allele of *SlRCM1*, and investigated its roles in chloroplast senescence and osmotic stress tolerance in tomato. We found that loss of *SlRCM1* function promotes premature chloroplast degradation during the later stages of leaf development and is associated with significantly elevated ABA levels and enhanced osmotic stress tolerance. Moreover, SlRCM1 interacts with SlNCED2, a key enzyme involved in ABA biosynthesis. Based on these findings, we propose that *SlRCM1* may be associated with ABA accumulation and osmotic stress responses, potentially through its interaction with SlNCED2.

## 2. Results

### 2.1. Comparative Phenotypic Characterization of the Wild-Type and rcm1t Mutant

**Figure 1:**
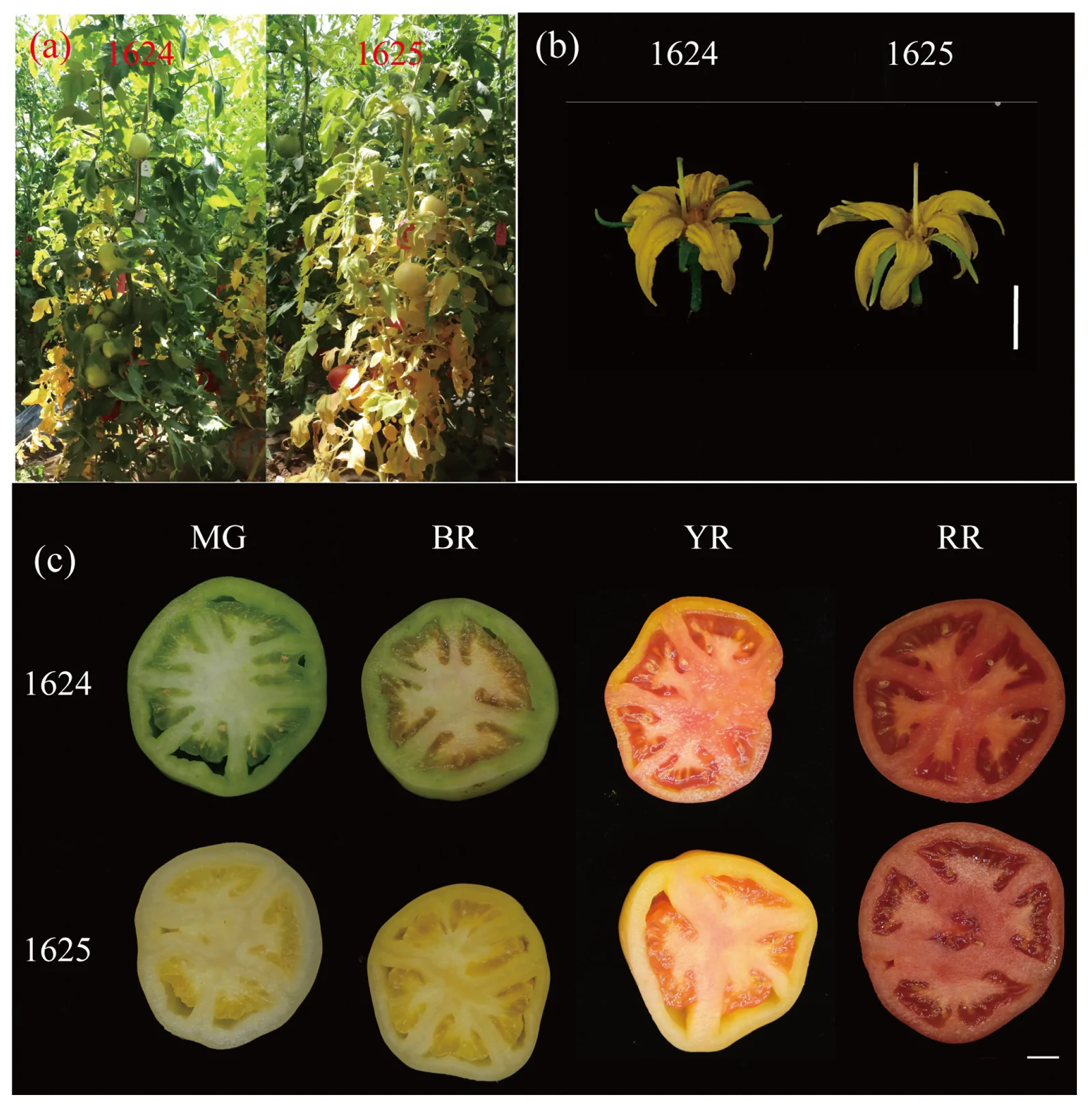
Phenotypic analysis of 1624 (*SlRCM1*) and 1625 (*rcm1t*). (**a**) Field-based phenotypic comparison of leaves between the tomato lines 1624 (*SlRCM1*) and 1625 (*rcm1t*). (**b**) Phenotypic comparison of flowers between 1624 (*SlRCM1*) and 1625 (*rcm1t*). (**c**) Comparative analysis of fruit development across Mature green (MG), Breaker (BR), Yellow ripe (YR), and Red ripe (RR) stages between 1624 (*SlRCM1*) and 1625 (*rcm1t*). The white bar indicates 1 cm.

**Figure 2:**
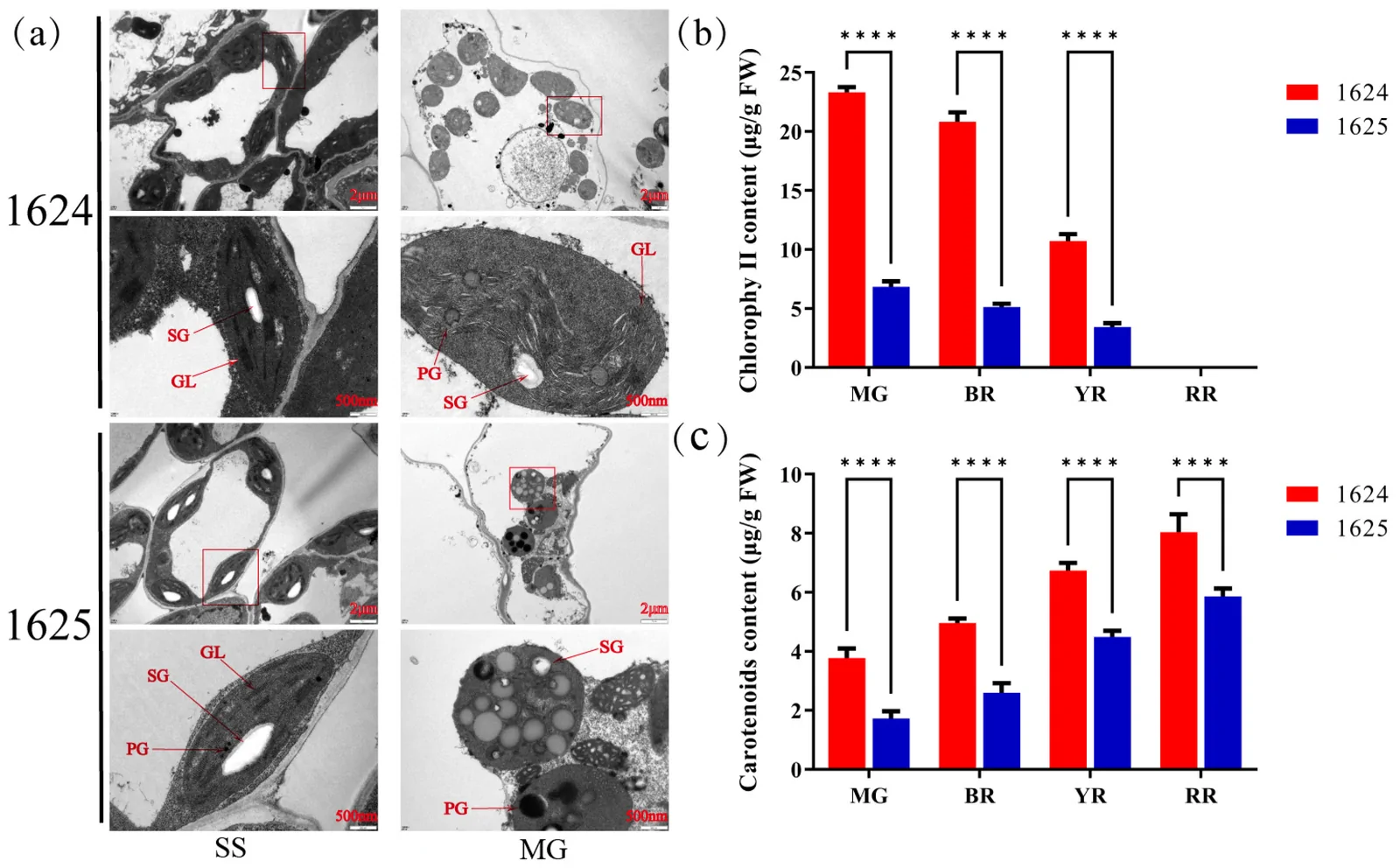
Comparative analysis of chloroplast ultrastructure and photosynthetic pigment content in 1624 (*SlRCM1*) and 1625 (*rcm1t*). (**a**) Comparative observation of the ultrastructure of chloroplasts in 1624 (*SlRCM1*) and 1625 (*rcm1t*). (**b**) Chlorophyll content comparison between 1624 (*SlRCM1*) and 1625 (*rcm1t*). (**c**) Comparison of carotenoid content. Asterisks indicate a significant difference (****, *p* < 0.0001).

Field investigation identified a chloroplast-associated premature senescence mutant (designated 1625, *rcm1t*) segregating from the advanced inbred line 1624 (*SlRCM1*). Phenotype characterization revealed tissue-specific premature chlorosis and yellowing—predominantly in chloroplast-rich organs including leaves, sepals, styles, and pericarp—with concomitant and significant reductions in chlorophyll and carotenoid contents (Figure 1 and Figure 2). However, before leaf chlorosis occurred, the chlorophyll and carotenoid contents in the leaves of 1625 did not show significant differences compared to those of 1624 (Figure S1).

Transmission electron microscopy (TEM) demonstrated that, in wild-type 1624 leaf mesophyll cells, chloroplasts were tightly anchored to the plasma membrane, maintained structural integrity, and exhibited well-organized, clearly resolved grana stacks (Figure 2a). In mutant 1625, chloroplasts in green leaf regions (seedling stage, SS) were morphologically indistinguishable from those in the wild type. However, in chlorotic and yellowed tissues (MG), grana lamellae (GL) progressively disassemble and lose structural integrity, concomitant with a significant increase in both the number and size of plastoglobules (PG); large electron-dense PG accumulate in aging plastids, coinciding with starch granule (SG) depletion and severe disruption of the thylakoid membrane system—collectively indicative of premature chloroplast senescence and structural collapse (Figure 2a).

**Figure 3:**
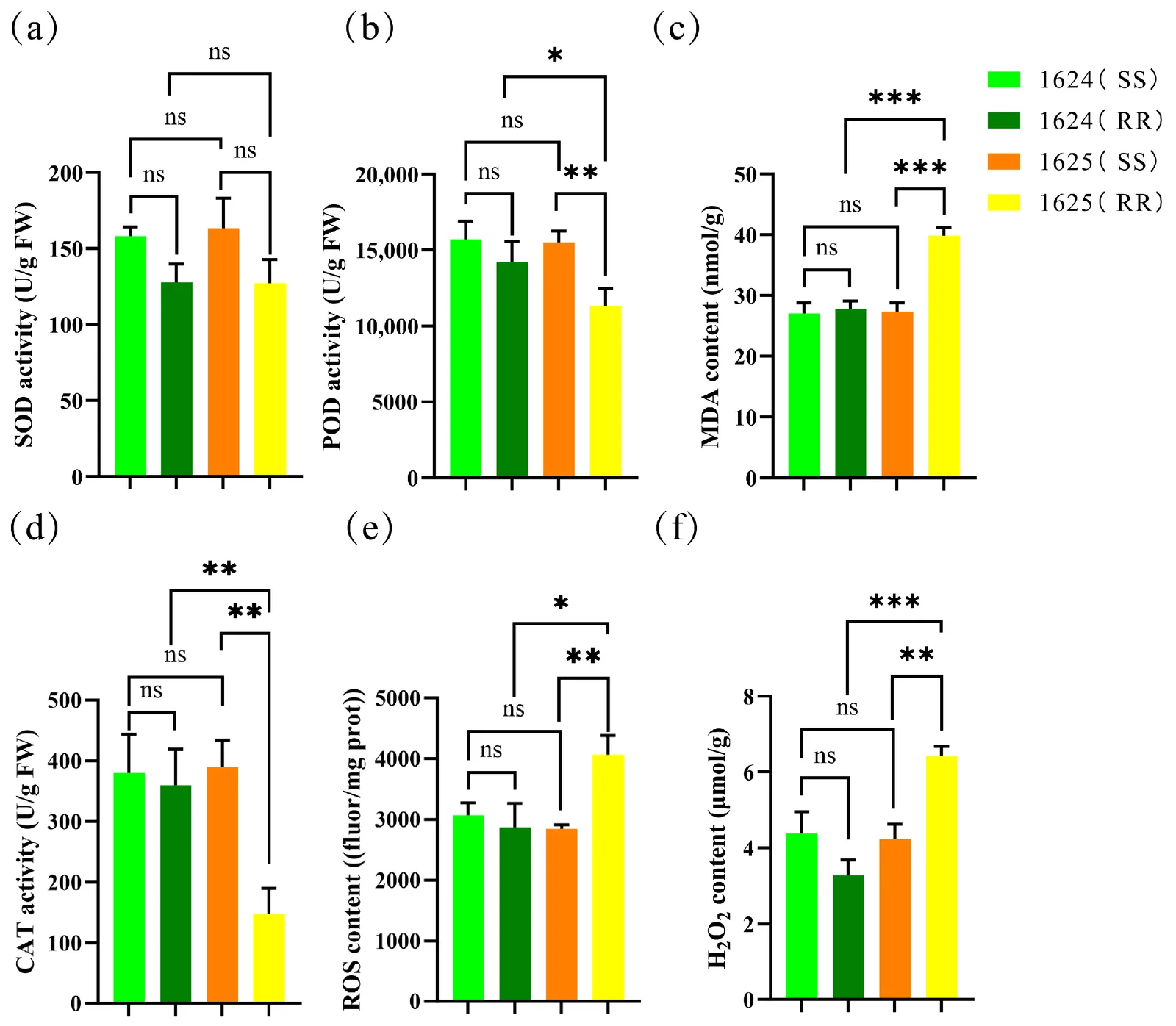
Determination of antioxidant enzyme activities and oxidative damage markers. (**a**) SOD activity assay. (**b**) POD activity assay. (**c**) MDA content assay. (**d**) CAT activity assay. (**e**) ROS content assay. (**f**) H_2_O_2_ content assay. Asterisks indicate a significant difference (*, *p* < 0.05; **, *p* < 0.01; ***, *p* < 0.001); ns indicates no significant difference.

To investigate the association between premature chloroplast senescence in line 1625 and key abiotic stress-related oxidative metabolites, we quantified established oxidative markers in leaves of 1624 and 1625 at the seedling stage (SS) and red-ripe stage (RR). Results revealed no significant differences in superoxide dismutase (SOD), peroxidase (POD), and catalase (CAT) activities, or in malondialdehyde (MDA), reactive oxygen species (ROS), and hydrogen peroxide (H_2_O_2_) levels, between 1624 and 1625 at the pre-chlorotic stage, before visible signs of chloroplast senescence became apparent (Figure 3). In the late stage of chloroplast premature senescence and chlorosis, the activities of POD and CAT, which are responsible for scavenging ROS, were significantly downregulated in line 1625 and were significantly lower than those in the control line 1624. Meanwhile, the levels of MDA, ROS, and H_2_O_2_, which are indicators of oxidative damage, were markedly upregulated and significantly higher than those in line 1624 (Figure 3).

### 2.2. Genetic Analysis and Fine-Mapping of rcm1t

A cross was performed with 1625 as the female parent and LA1589 as the male parent. All F_1_ progeny exhibited normal chloroplast development and showed no signs of premature senescence or chlorosis. In the F_2_ generation, segregation for chloroplast premature senescence was observed: 634 plants displayed the wild-type phenotype, whereas 217 exhibited the mutant phenotype. The observed ratio (634:217 ≈ 2.92:1) did not significantly deviate from the expected 3:1 Mendelian ratio (χ^2^ = 0.11; df = 1; critical value χ^2^_0.05,1_ = 3.84), supporting the conclusion that the chloroplast premature senescence phenotype in the 1625 mutant is conferred by a single recessive gene.

**Figure 4:**
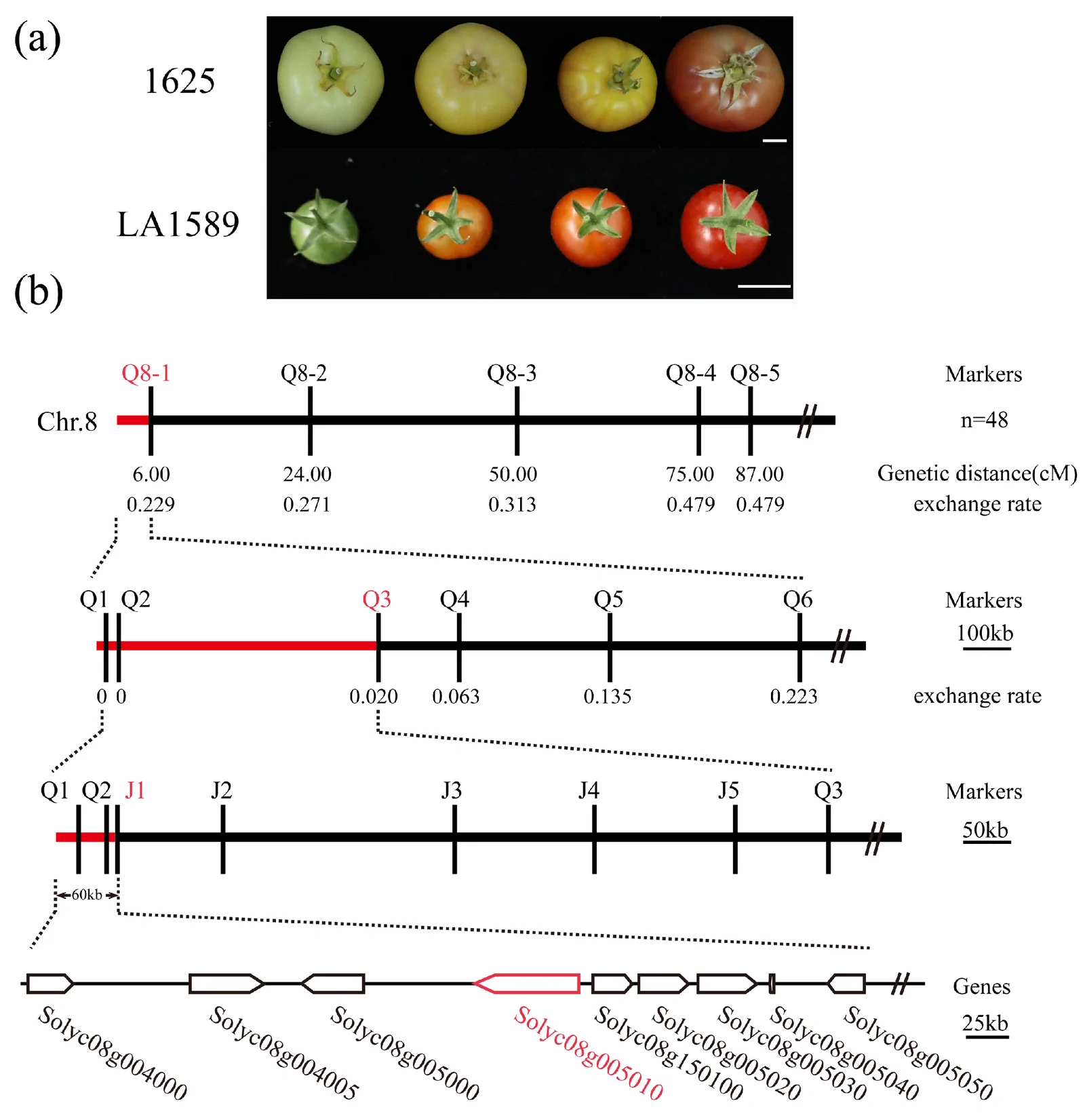
Fine-mapping of *rcm1t*. (**a**) Parental fruit phenotypic traits. (**b**) Genetic and physical mapping and fine-mapping of the *rcm1t* locus. The white bar indicates 1 cm.

**Figure 5:**
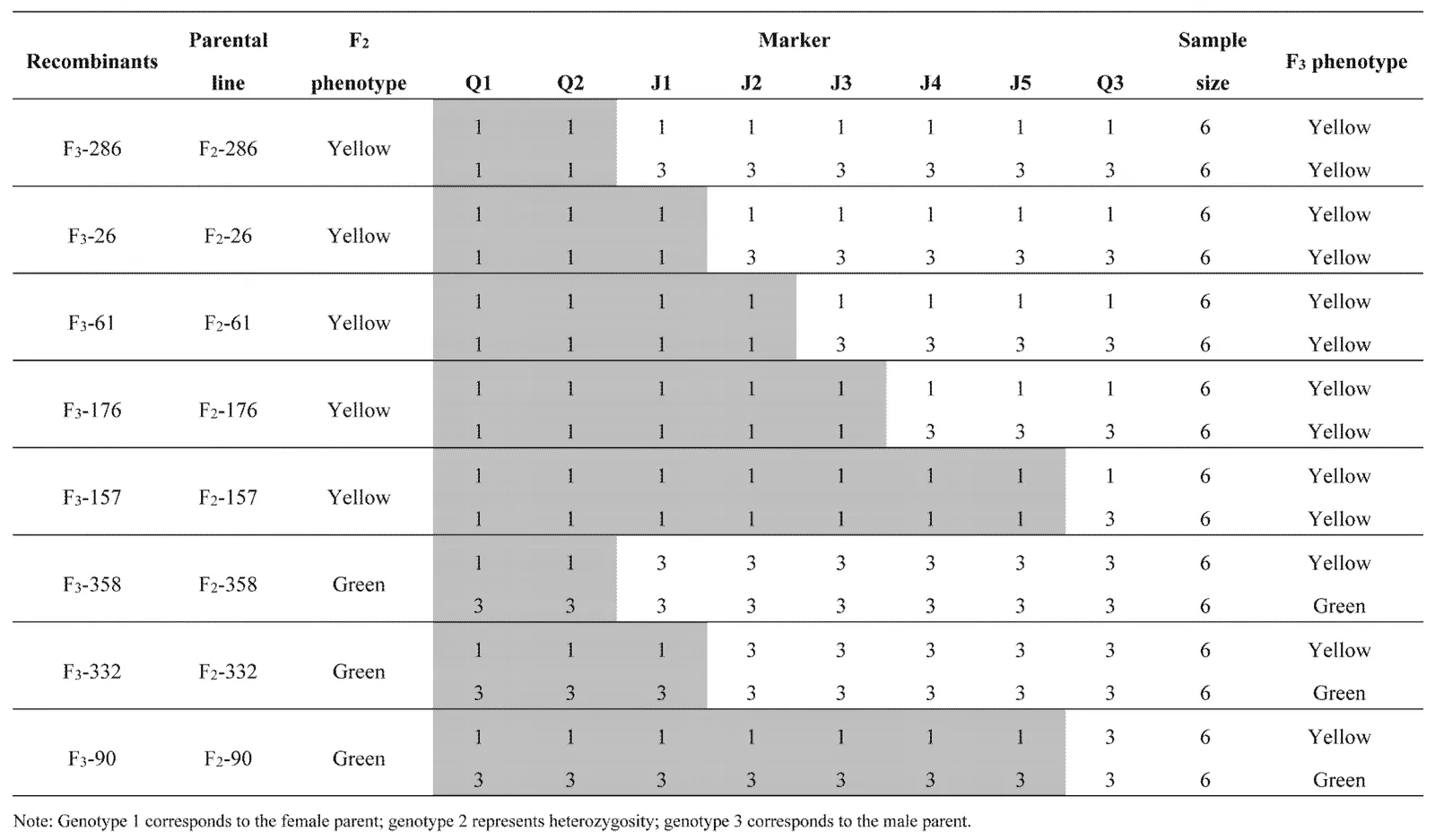
Genotypic and phenotypic analysis of F_2_/F_3_ partial recombinants.

For a qualitative trait governed by a single recessive gene, we applied population segregation analysis. On each chromosome, 4–6 polymorphic molecular markers were evenly distributed for genome-wide screening. Initial mapping was conducted using DNA pools from 48 F_2_ individuals exhibiting the recessive phenotype. Recombination frequency analysis revealed a progressive decline from downstream to upstream along chromosome 8, with marker Q8-1 showing the lowest recombination frequency (0.229), suggesting proximity to the causal locus. To refine the mapping interval, six additional markers were developed in the upstream region of Q8-1. Consistent with this trend, recombination frequencies decreased further upstream: markers Q1 and Q2 exhibited zero recombination, indicating tight linkage to the *rcm1t* locus. Notably, marker Q3 (located at physical position 822,038 bp) displayed a recombination frequency of only 0.02, thereby delimiting the *rcm1t* gene to a preliminary interval spanning 0–0.82 Mb upstream on chromosome 8. To further narrow the candidate interval of the target gene, we saturated the region upstream of marker Q3 with additional molecular markers and integrated genotypes from recombinant progeny with phenotypic analysis, thereby fine-mapping the *rcm1t* locus to a 60-kb interval flanked by markers Q1 and J1 (Figure 4 and Figure 5).

### 2.3. Analysis and Functional Characterization of Candidate Gene

According to the ITAG4.0 genome annotation, nine candidate genes reside within the mapped interval. Comparative analysis of their expression profiles and coding sequences identified *Solyc08g005010* (*SlRCM1*) as the sole candidate exhibiting both differential expression and nonsynonymous coding variants (Table 1 and Figure S2).

**Figure 6:**
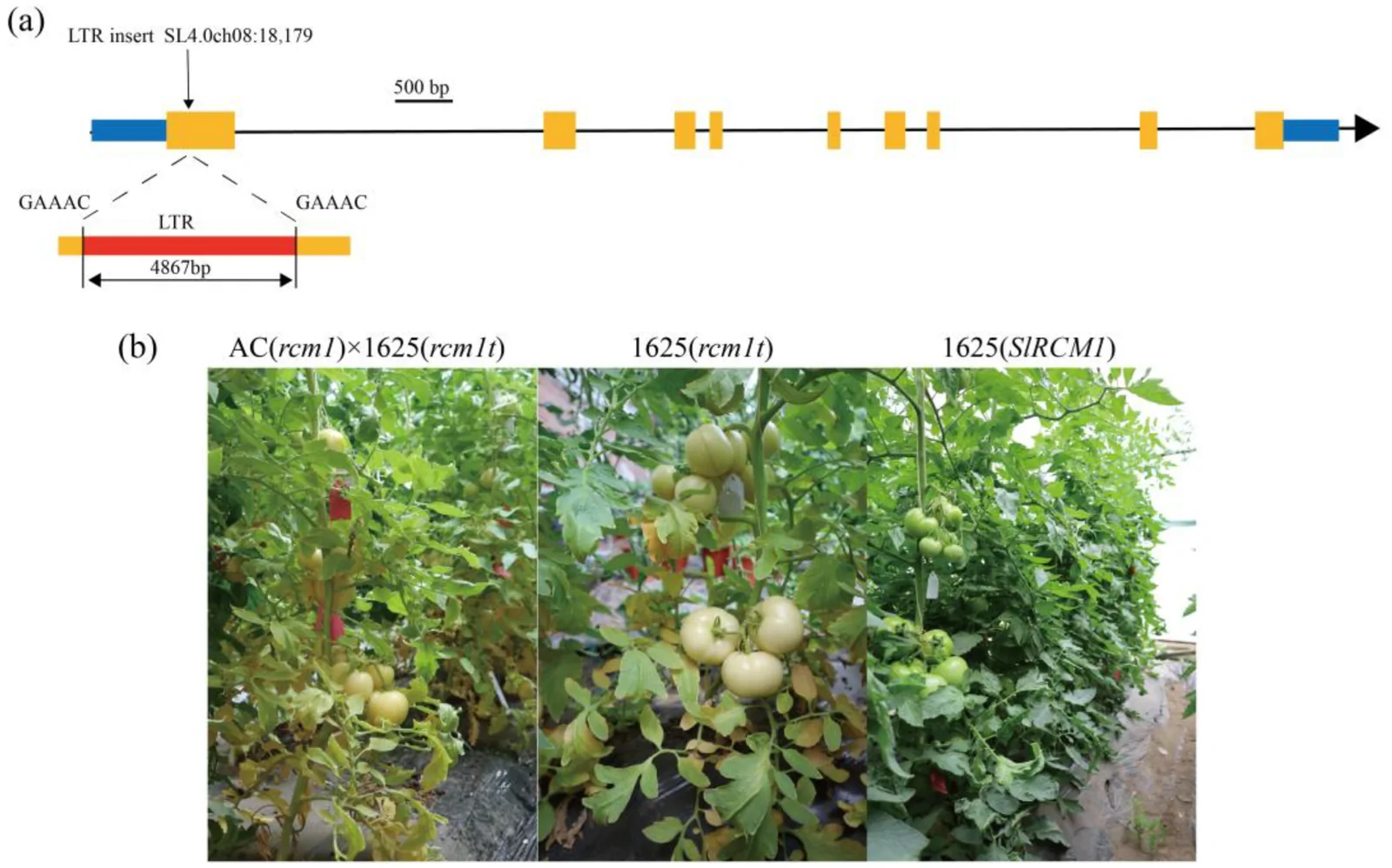
Analysis and functional characterization of the candidate gene. (**a**) Structure of the *SlRCM1* gene according to ITAG4.0 and the structure of the LTR retrotransposon inserted in the first exon. (**b**) Allelic testing and functional complementation assay.

Further sequence analysis identified a 4867-bp long terminal repeat (LTR) retrotransposon insertion immediately downstream of the 66th nucleotide in the first exon of *Solyc08g005010* (Figure S3). This insertion disrupted the reading frame and induced a 73-bp deletion in the coding sequence, followed by the introduction of a TGA stop codon—leading to premature translational termination and a severely truncated protein product (Figure 6a). *Solyc08g005010* is annotated as encoding a CaaX amino-terminal protease family protein. CaaX proteases generally recognize prenylated proteins containing a C-terminal CaaX motif and remove the terminal -aaX tripeptide [18]. Prior genetic studies in tomato demonstrated that ethyl methanesulfonate (EMS)-induced loss-of-function alleles of this gene cause premature chloroplast senescence and leaf chlorosis. Therefore, *Solyc08g005010* is designated as the causal candidate gene for *rcm1t*.

An allelic complementation test between the 1625 (*rcm1t*) line and the EMS-induced *rcm1* mutant showed that all F_1_ progeny exhibited the premature chloroplast senescence phenotype, confirming that *rcm1t* is allelic to *rcm1*. To validate functional equivalence, we constructed an *SlRCM1* expression vector under the control of its native 3.5-kb promoter and introduced it into the 1625 (*rcm1t*) background. Three independent *SlRCM1* transgenic lines completely rescued the chloroplast premature senescence phenotype. This unambiguous genetic complementation confirms that *rcm1t* is a naturally occurring loss-of-function allele of *SlRCM1* (Figure 6b and Figure S4).

### 2.4. Comparative RNA-Seq Analysis of SlRCM1 and rcm1

**Figure 7:**
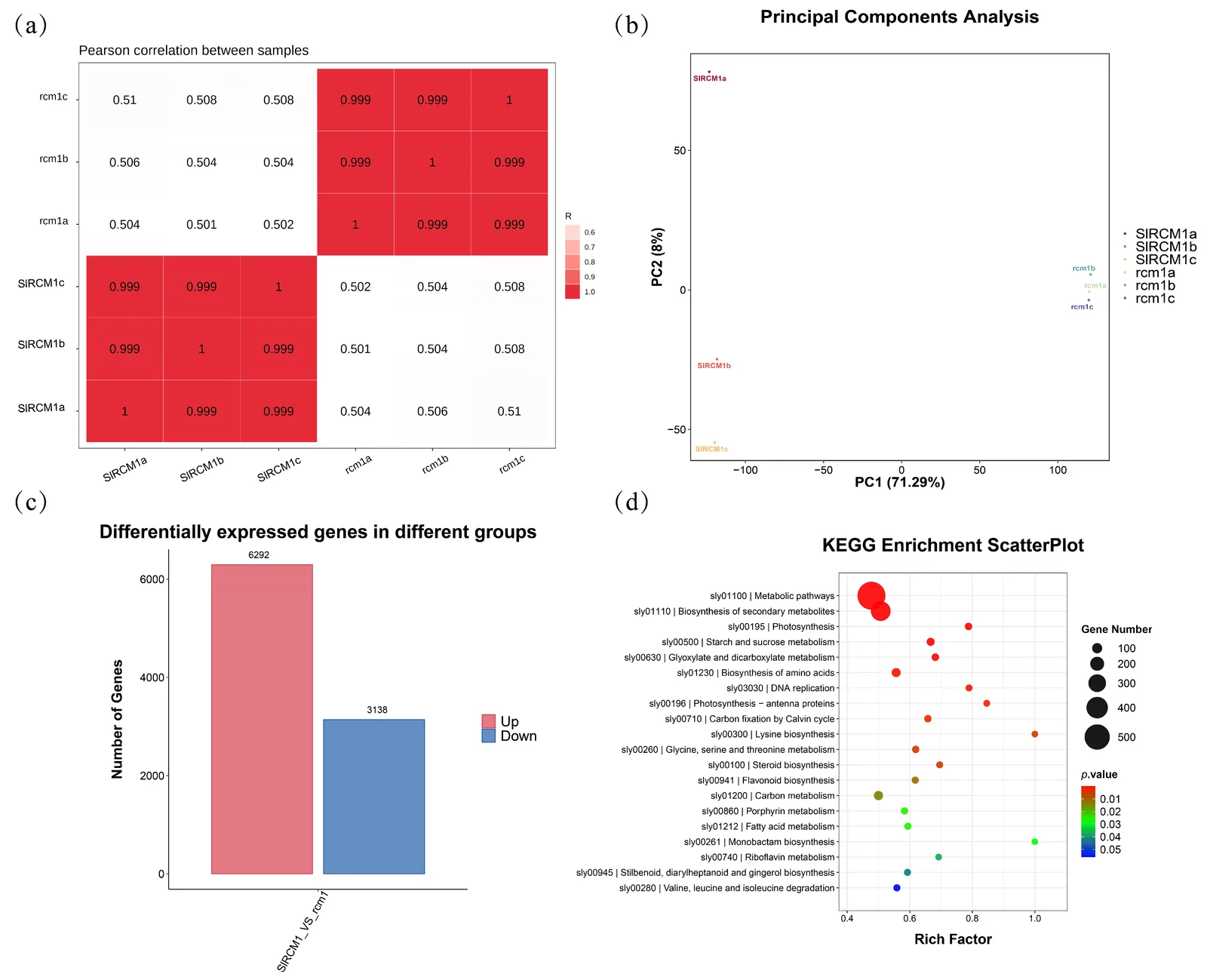
Comparative RNA-seq analysis of *SlRCM1* and *rcm1*. (**a**) Sample correlation analysis. (**b**) Principal component analysis of RNA-seq data. (**c**) Number of differentially expressed genes. (**d**) Functional enrichment analysis of differentially expressed genes.

To further elucidate the molecular mechanism by which *SlRCM1* regulates chloroplast senescence in tomato, we performed RNA sequencing (RNA-seq) analysis on leaves of the wild-type line Ailsa Craig (AC) (*SlRCM1*) and its CRISPR/Cas9-generated loss-of-function mutant AC (*rcm1*). Sequencing yielded approximately 205 million high-quality paired-end reads, with each biological replicate generating 30–40 million reads. On average, 95% of reads were uniquely mapped to the ITAG4.0 tomato reference genome (Table S1). High inter-replicate correlation coefficients (Pearson *r* > 0.95) and tight clustering of biological replicates in principal component analysis collectively demonstrate excellent technical reproducibility and data robustness—confirming that the transcriptomic dataset is of high quality and suitable for downstream differential expression and functional analyses (Figure 7a,b).

Comparative transcriptomic analysis between wild type and *rcm1* leaves identified 9430 differentially expressed genes (DEGs), including 6154 upregulated and 3276 downregulated genes (Figure 7c). Kyoto Encyclopedia of Genes and Genomes (KEGG) pathway enrichment analysis of these DEGs revealed significant overrepresentation of genes involved in primary metabolism and secondary metabolism, indicating that loss of *SlRCM1* function is associated with widespread transcriptional alterations in tomato leaves (Figure 7d).

**Figure 8:**
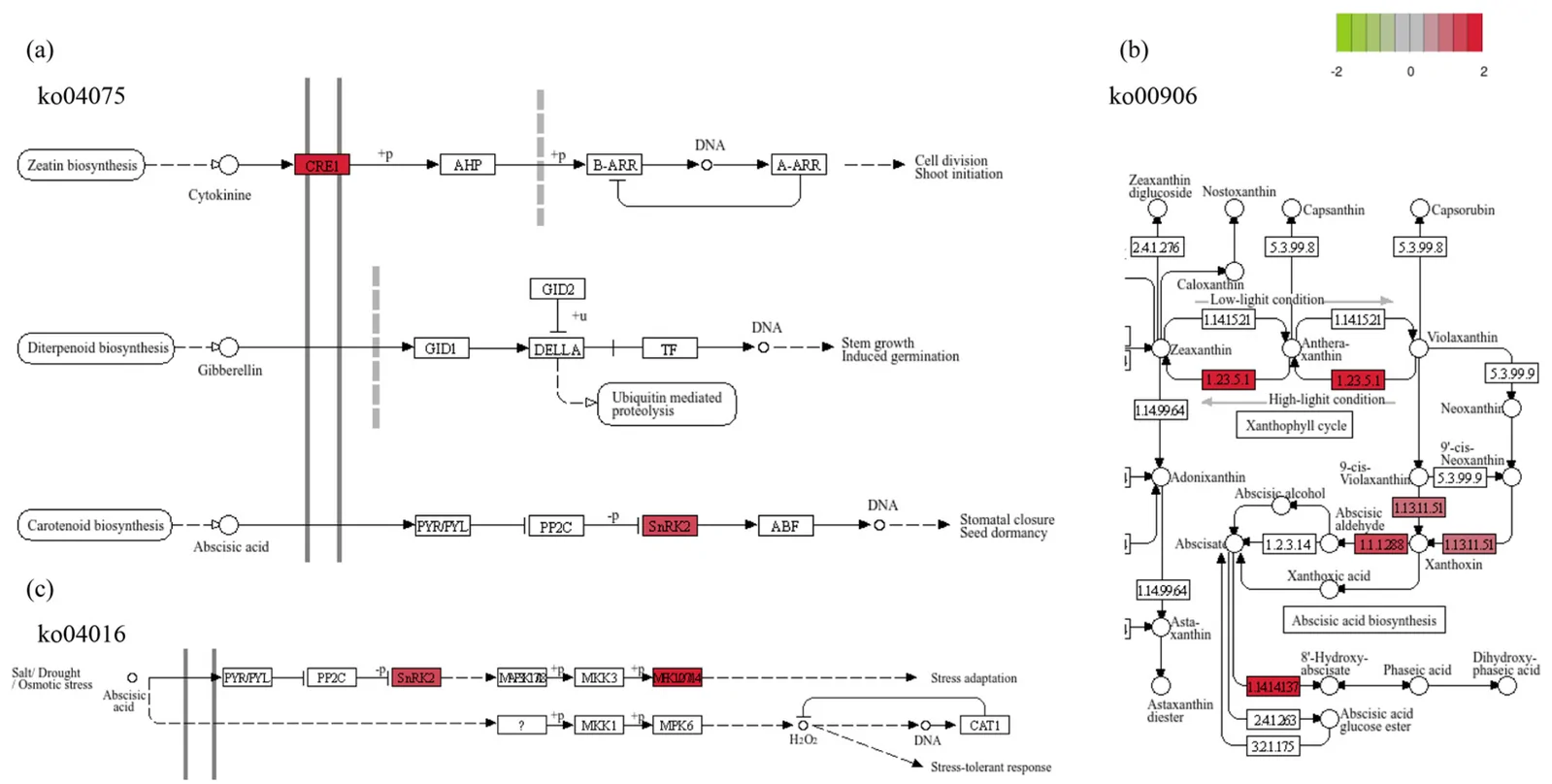
KEGG pathway enrichment of DEGs. (**a**) Partially enriched KEGG pathway ko04075. (**b**) Partially enriched KEGG pathway ko00906. (**c**) Partially enriched KEGG pathway ko04016.

Further analysis of these DEGs revealed, in addition to significant enrichment in pathways related to aging regulation (ko00480, ko00860, ko00360), that the ABA-related pathways ko04075, ko00906, and ko04016 were also enriched among the highly upregulated DEGs, indicating that ABA-related transcriptional processes were altered in *rcm1* (Figure 8 and Figures S5–S7).

### 2.5. SlRCM1 Interacts with SlNCED2 and Is Associated with ABA Accumulation

**Figure 9:**
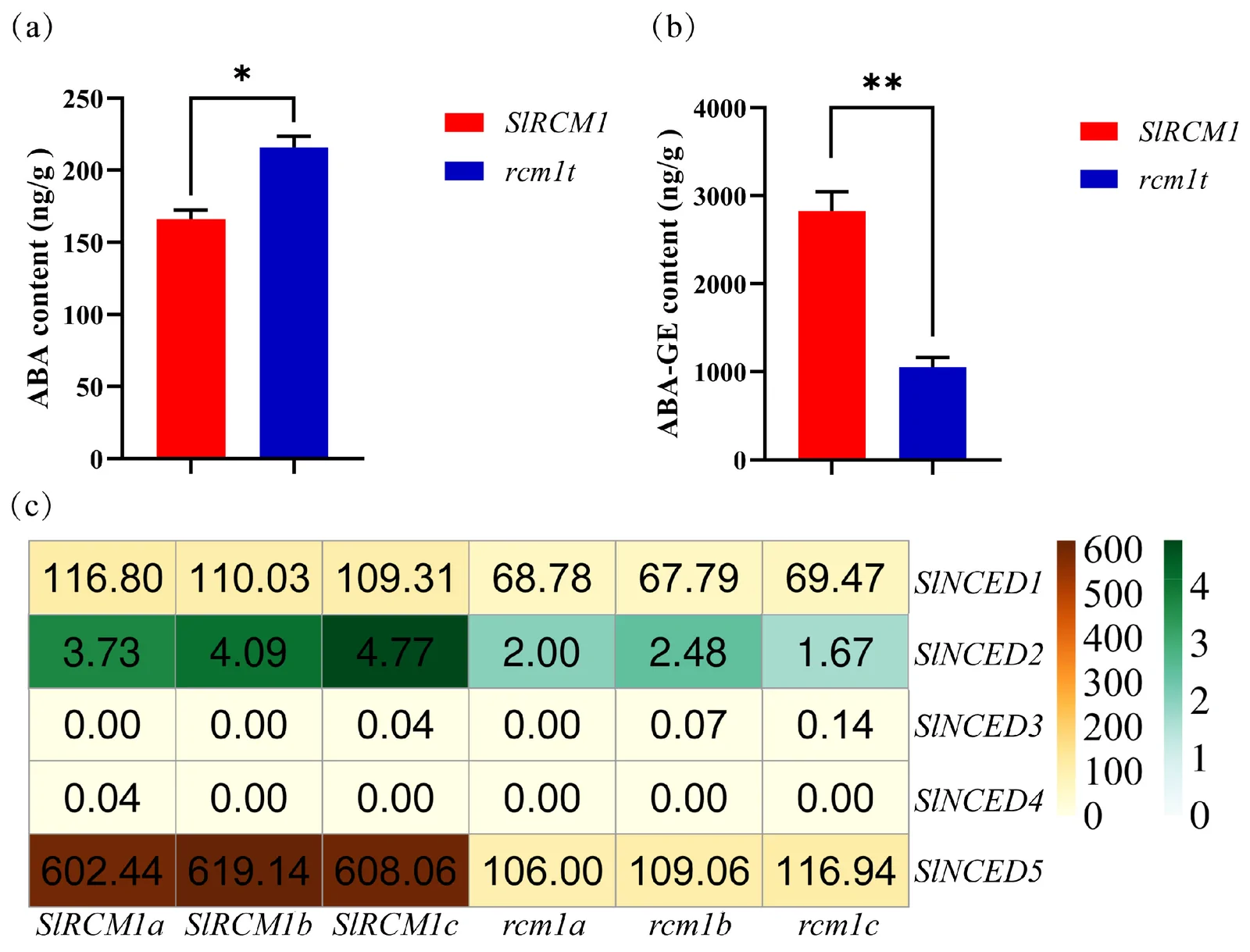
Quantification of ABA and ABA-GE and expression analysis of *SlNCED* family genes. (**a**) ABA content in leaves of line 1624 (*SlRCM1*) and line 1625 (*rcm1t*). (**b**) ABA-GE content in leaves of line 1624 (*SlRCM1*) and line 1625 (*rcm1t*). (**c**) Heatmap showing the transcript levels of *SlNCED* family genes based on RNA-seq analysis of AC (*SlRCM1*) and the CRISPR/Cas9-generated AC (*rcm1*) mutant. Asterisks indicate a significant difference (*, *p* < 0.05; **, *p* < 0.01). Heatmap values represent fragments per kilobase of transcript per million mapped reads (FPKM).

Transcriptome analysis indicated that loss of *SlRCM1* function was associated with alterations in ABA biosynthesis-and homeostasis-related pathways. To further examine whether these transcriptional changes were accompanied by altered ABA homeostasis, we quantified ABA and its biologically inactive glucose-conjugated form, β-D-glucopyranosyl abscisate (ABA-GE), in leaf tissues of 1624 (*SlRCM1*) and 1625 (*rcm1t*). The results showed that the ABA levels were significantly lower in *SlRCM1* (166.18 ± 5.01 ng/g) than in *rcm1t* (215.73 ± 5.49 ng/g), whereas ABA-GE levels were significantly higher in *SlRCM1* (2825.90 ± 179.93 ng/g) than in *rcm1t* (1052.04 ± 92.71 ng/g) (Figure 9). These contrasting changes in ABA and ABA-GE levels indicate that loss of *SlRCM1* function is associated with altered ABA homeostasis, supporting a potential role for SlRCM1 in the regulation of ABA metabolism. Previous studies in maize have demonstrated that ZmCAAX, a homolog of SlRCM1, physically interacts with ZmNCED3 and promotes its degradation, with loss of ZmCAAX function being associated with increased ZmNCED3 protein abundance and ABA accumulation [15]. Transcriptome sequencing identified three *SlNCED* genes expressed in tomato leaves. Notably, the transcript levels of all three *SlNCED* genes were significantly lower in AC (*rcm1*) than in AC (*SlRCM1*) (*p* < 0.01) (Figure 9c). Given the previously reported interaction between ZmCAAX and ZmNCED3 in maize, we therefore investigated whether SlRCM1 could interact with SlNCED proteins in tomato.

**Figure 10:**
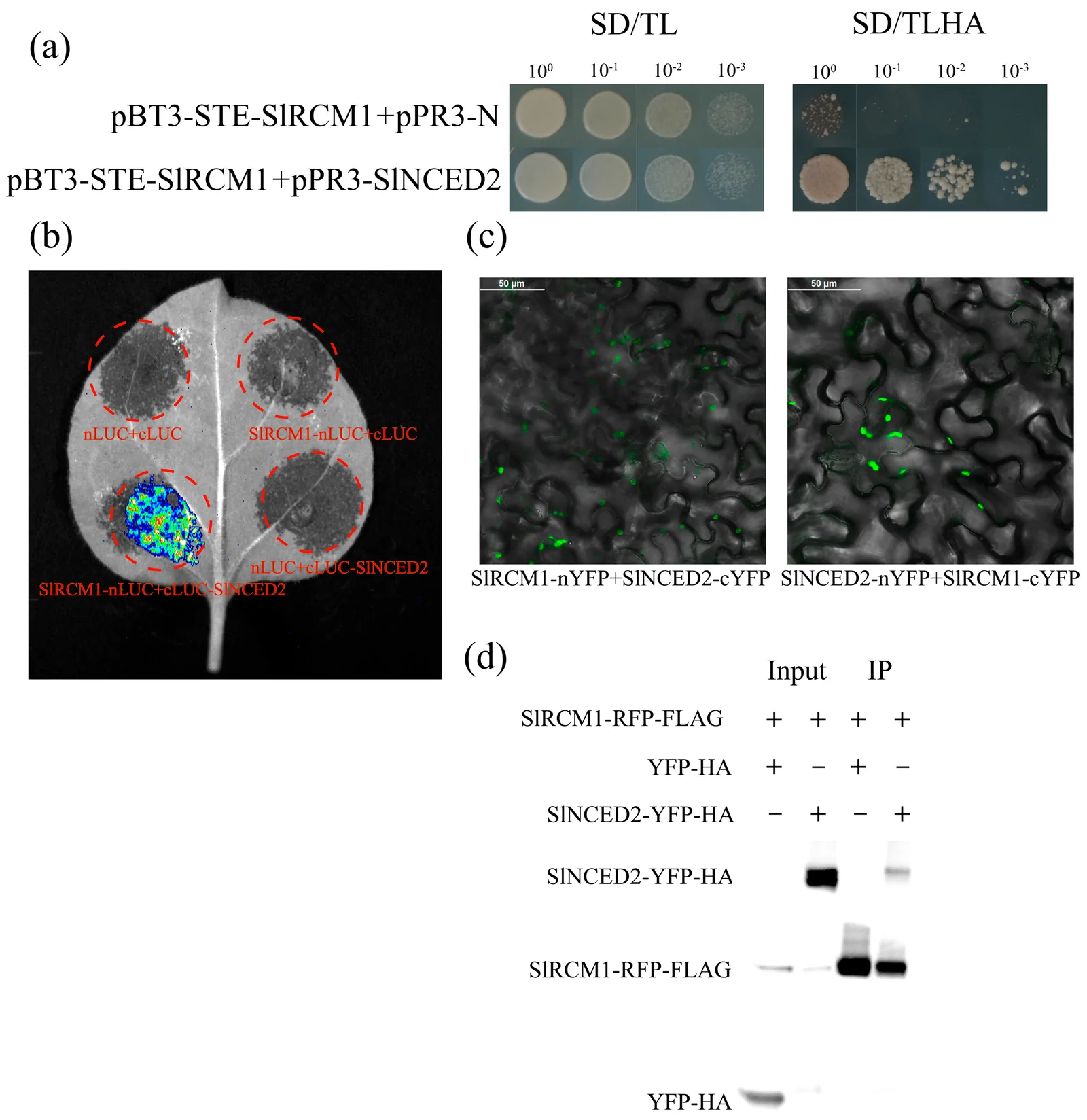
Verification of the interaction between SlRCM1 and SlNCED2. (**a**) The Y2H assay. (**b**) The LCI assay. (**c**) The BiFC assay. (**d**) The Co-IP assay.

To examine the interaction between SlRCM1 and three SlNCED proteins—SlNCED1, SlNCED2, and SlNCED5—we performed a yeast two-hybrid (Y2H) assay and identified SlNCED2 as the only SlNCED protein that interacts with SlRCM1 (Figure 10a and Figure S9). This interaction was further corroborated by a luciferase complementation imaging (LCI) assay: co-expression of SlRCM1-nLUC and cLUC-SlNCED2 produced a clearly detectable luminescence signal compared with the negative controls (Figure 10b). Subcellular localization analysis showed that SlNCED2 was predominantly localized to chloroplasts (Figure S8). Consistent with this localization, bimolecular fluorescence complementation (BiFC) assays revealed strong yellow fluorescent protein (YFP) fluorescence specifically localized to chloroplasts in *N. benthamiana* leaves co-expressing SlRCM1-n/cYFP and SlNCED2-c/nYFP, indicating chloroplast-localized interaction (Figure 10c). Finally, co-immunoprecipitation (Co-IP) assays demonstrated that SlRCM1-RFP-FLAG efficiently co-precipitated SlNCED2-YFP-HA from plant extracts, whereas no signal was detected for YFP-HA alone, providing biochemical evidence of a specific, in planta SlRCM1–SlNCED2 complex (Figure 10d).

### 2.6. rcm1t Confers Osmotic Stress Tolerance in Tomato

**Figure 11:**
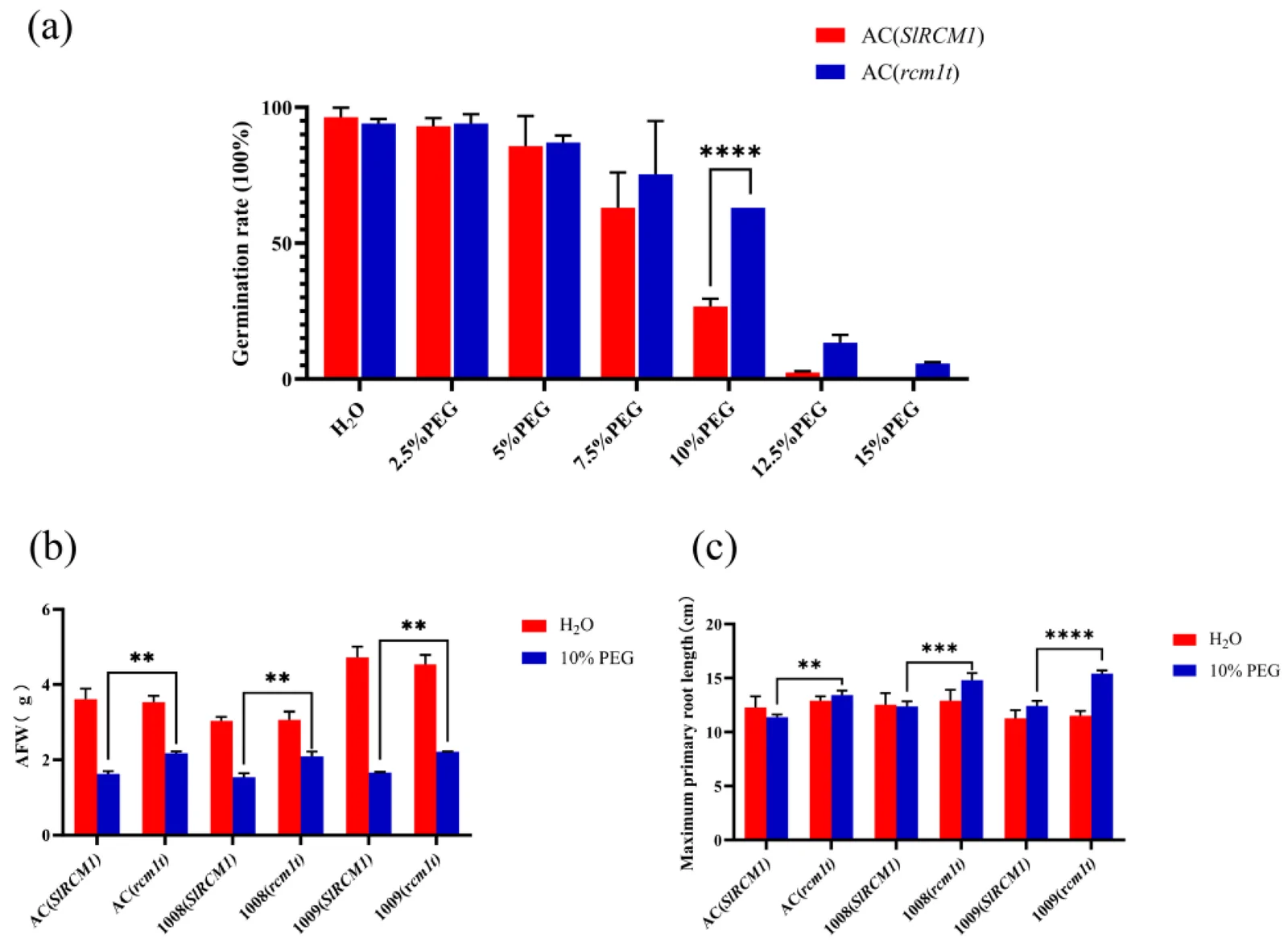
Analysis of *rcm1t*-mediated osmotic-stress tolerance. (**a**) Germination rates of AC (*SlRCM1*) and AC (*rcm1t*) under increasing PEG-6000 concentrations. (**b**) Measurement of aboveground fresh weight in tomato plants. (**c**) Measurement of maximum primary root length in tomato plants. Asterisks indicate a significant difference (**, *p* < 0.01; ***, *p* < 0.001; ****, *p* < 0.0001).

To functionally characterize SlRCM1’s role in tomato osmotic stress tolerance, we developed near-isogenic lines (NILs) carrying the *rcm1t* allele through backcrossing into three elite recurrent parents—AC, 1008, and 1009. In the AC background, seed germination progressively declined with increasing polyethylene glycol 6000 (PEG-6000) concentration. Two-way ANOVA revealed significant effects of genotype and PEG concentration, as well as a significant genotype × PEG concentration interaction (*p* = 0.00167), indicating that the effect of *rcm1t* on germination depended on stress intensity (Table S3). Šídák-adjusted comparisons showed the strongest genotypic difference at 10% PEG-6000, where AC (*rcm1t*) exhibited a significantly higher germination rate than AC (*SlRCM1*) (63.0% vs. 26.7%, adjusted *p* < 0.0001; Figure 11a). Therefore, 10% PEG-6000 was used for subsequent seedling assays.

The effects of *rcm1t* on aboveground fresh weight (AFW) and maximum primary root length were further evaluated in the three NIL backgrounds using three-way ANOVA with genetic background, genotype, and treatment as factors. Significant genotype × treatment interactions were detected for both AFW and primary root length (*p* < 0.0001), whereas the background × genotype × treatment interactions were not significant (Table S3). Under control conditions, no significant genotypic differences were observed. In contrast, under 10% PEG-6000, *rcm1t* significantly increased AFW by 32.9–35.6% and primary root length by 18.2–23.9% across all three genetic backgrounds (Figure 11b,c). Significant background × treatment interactions were also detected, indicating that the overall response to PEG differed among genetic backgrounds.

**Figure 12:**
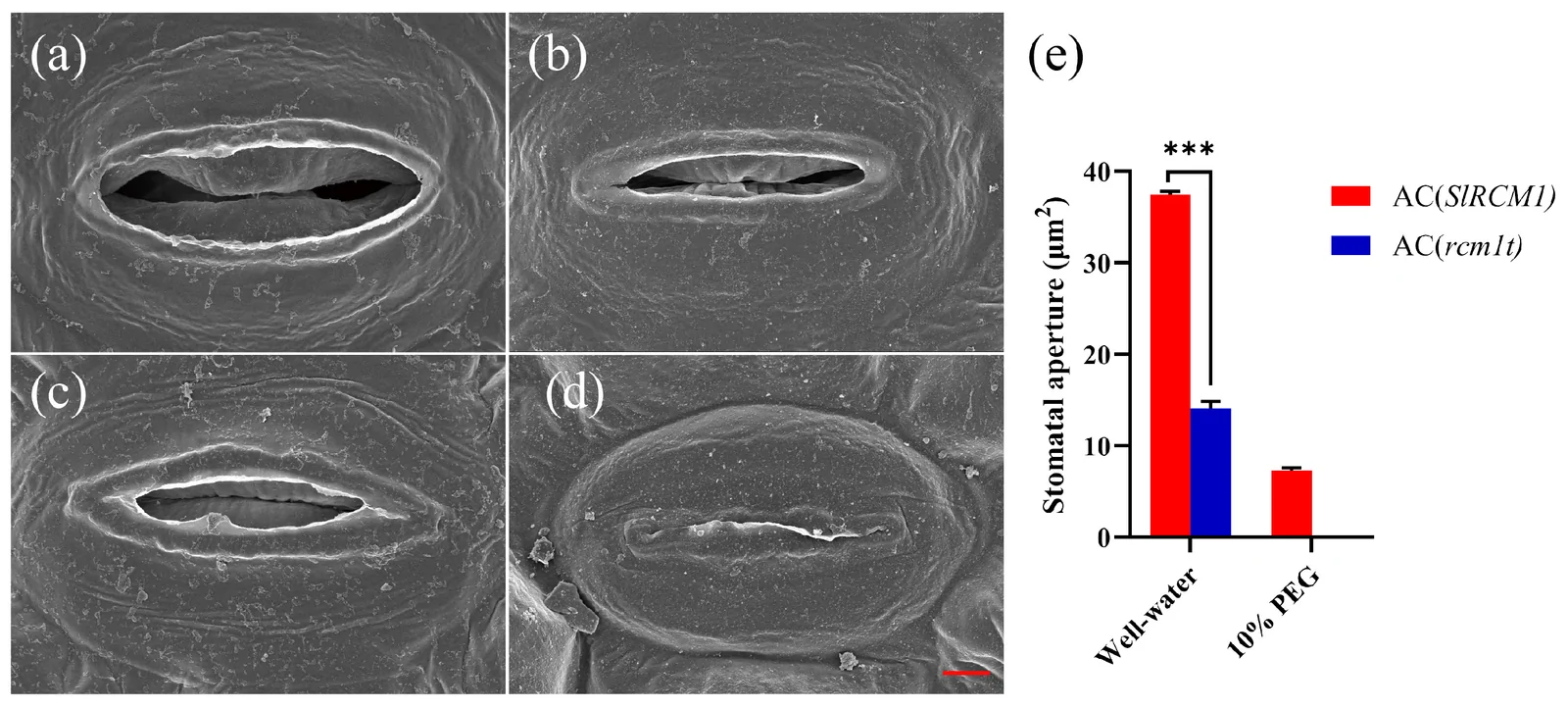
Stomatal aperture of AC (*SlRCM1*) and AC (*rcm1t*) under well-watered and osmotic-stress conditions. (**a**) Stomatal aperture of AC (*SlRCM1*) under well-watered conditions. (**b**) Stomatal aperture of AC (*rcm1t*) under well-watered conditions. (**c**) Stomatal aperture of AC (*SlRCM1*) under osmotic-stress conditions. (**d**) Stomatal aperture of AC (*rcm1t*) under osmotic-stress conditions. (**e**) Stomatal aperture under different conditions. The red bar indicates 2 μm. Asterisks indicate a significant difference (***, *p* < 0.001).

Furthermore, scanning electron microscope (SEM) analysis revealed that stomatal aperture in AC (*rcm1t*) was significantly smaller than that in AC (*SlRCM1*) under both well-watered and osmotic-stress conditions (Figure 12e).

## 3. Discussion

Chloroplasts are essential organelles in plant cells, functioning not only as the primary site of photosynthesis but also as central coordinators of metabolic and signaling networks [22,23]. Beyond energy conversion, they integrate ROS homeostasis, phytohormone biosynthesis, and senescence regulation—processes that collectively govern plant growth, developmental progression, and adaptive responses to abiotic and biotic stresses [22,23,24,25,26,27]. During leaf senescence, chloroplasts serve as the earliest cellular sensors of senescence signals and trigger programmed disassembly [28,29,30]. Key hallmarks of chloroplast aging encompass a marked decline in thylakoid stacking (reduced grana lamellae number), structural swelling of grana, changes in starch granules and plastoglobules, and compromised integrity of the chloroplast envelope [28,31]. Phenotypic comparison of leaves between mutant 1625 and its wild-type counterpart 1624, together with transmission electron microscopy analysis of chloroplast ultrastructure at different developmental stages, showed that chloroplasts in the green tissues of mutant 1625 were morphologically comparable to those of the wild type and maintained intact ultrastructure. In contrast, chloroplasts in chlorotic tissues exhibited progressive senescence-associated ultrastructural deterioration, including chloroplast envelope disruption and thylakoid disassembly. These observations indicate that the chlorotic phenotype of mutant 1625 is primarily attributable to premature chloroplast senescence rather than defects in early chloroplast development or chlorophyll biosynthesis [12,31].

In multiple regulatory pathways involving chloroplasts, their coordinated regulation of ROS homeostasis, ABA biosynthesis, and senescence processes is particularly critical [32,33]. These three interdependent processes converge functionally on the chloroplast, establishing it as a key regulatory hub within a multidimensional signaling network [34,35]. As the predominant intracellular site for both ROS generation and scavenging, chloroplast structural and functional integrity is a decisive determinant of ROS production, accumulation, and the fidelity of downstream signal transduction [36,37,38,39]. The thylakoid membrane is the predominant site of ROS generation within chloroplasts [36]. Under physiological conditions, controlled ROS production arises primarily from electron leakage at Photosystem I (PSI) and Photosystem II (PSII), integral components of the photosynthetic electron transport chain [40]. At basal concentrations, ROS function as essential redox signaling molecules, regulating chloroplast biogenesis, thylakoid differentiation, and the homeostatic maintenance of photosynthetic electron flux [35]. In contrast, under abiotic stress (high light irradiance, drought, or temperature extremes) or upon structural compromise (e.g., thylakoid unstacking, envelope integrity loss), ROS production surges markedly [41]. This shift from low-level signaling to excessive accumulation triggers oxidative damage, thereby accelerating chloroplast disassembly and compromising cellular redox homeostasis [38]. To prevent irreversible oxidative damage resulting from ROS overaccumulation, plants deploy a dual-tiered ROS detoxification system [39]. First, non-enzymatic photoprotective mechanisms dissipate excess excitation energy as heat, thereby minimizing over-reduction in the photosynthetic electron transport chain and suppressing ROS generation at its origin [42]. Second, enzymatic antioxidant defenses rely on antioxidant enzymes such as SOD, CAT, and POD to sequentially convert superoxide anions and hydrogen peroxide into water and oxygen via cascade enzymatic reactions, thus efficiently eliminating ROS [39]. Together, these observations indicate that premature chloroplast senescence in mutant 1625 is associated with disrupted oxidative homeostasis. Relative to wild-type 1624, aging tissues of the mutant exhibited significantly elevated ROS, H_2_O_2_, and MDA levels, accompanied by reduced POD and CAT activities. These changes are consistent with increased oxidative damage and may contribute to the accelerated structural deterioration of chloroplasts.

Moreover, chloroplasts are the initial site for ABA synthesis, and the generation of its precursor at the thylakoid membrane represents an irreversible rate-limiting step in the entire biosynthetic pathway, which is catalyzed by NCED [22,43,44]. Therefore, the functional status of chloroplasts is a key factor influencing the ABA synthesis pathway. In this study, transcriptome profiling of AC (*SlRCM1*) and AC (*rcm1*) mutants revealed significant enrichment of DEGs associated with ABA-related processes. Separately, quantification of ABA and ABA-GE in lines 1624 (*SlRCM1*) and 1625 (*rcm1t*) showed significantly higher ABA levels and lower ABA-GE levels in *rcm1t* than in its wild-type counterpart. Across the two independent experiments, the opposite trends in *SlNCED* transcript abundance and ABA accumulation observed between the respective wild-type and mutant materials suggest that the elevated ABA level associated with loss of *SlRCM1* function cannot be readily explained by transcriptional activation of ABA biosynthetic genes alone, raising the possibility that *SlRCM1* may influence ABA accumulation through additional regulatory mechanisms, potentially at the post-transcriptional or protein level. Alternatively, the transcriptional changes observed in the AC (*SlRCM1*) line may reflect feedback responses associated with the maintenance of ABA homeostasis. Given that loss of *SlRCM1* also induces premature chloroplast senescence and extensive downstream physiological changes, it remains unclear whether the altered ABA accumulation in *rcm1t* is a direct consequence of *SlRCM1*-dependent regulation or an indirect effect of senescence-associated metabolic and signaling changes.

Previous reports indicated that ZmCAAX, the homolog of the SlRCM1 protein in *Z. mays*, interacts with ZmNCED3 to reduce its promotion of ABA synthesis, thereby decreasing ABA production. When ZmCAAX is mutated, it loses the ability to interact with ZmNCED3, indirectly increasing ABA synthesis and accumulation, and significantly enhancing plant drought tolerance [15]. In this study, we also found that the transcription levels of *SlNCED* gene family members were significantly altered after the *SlRCM1* mutation in tomato. Subsequent Y2H, BiFC, LCI, and Co-IP assays confirmed the interaction between tomato SlRCM1 and SlNCED2 in chloroplasts. Considering that ABA content in tomato leaves significantly increases upon *SlRCM1* loss of function, we hypothesize that the interaction between SlRCM1 and SlNCED2 may be associated with ABA homeostasis; whether SlRCM1 restricts ABA accumulation by modulating SlNCED2 stability, abundance, or catalytic activity remains to be determined.

As a central phytohormonal regulator of drought responses, ABA promotes stomatal closure to minimize transpirational water loss [45]. In the present study, *rcm1t* exhibited enhanced osmotic stress tolerance, and stomatal aperture in the AC (*rcm1t*) line was significantly smaller than that in the wild-type control under both well-watered and osmotic stress conditions. Together with the significantly elevated ABA levels observed in *rcm1t*, these observations are compatible with a working model in which altered ABA accumulation may be associated with reduced stomatal aperture and enhanced osmotic stress tolerance; however, the causal relationships among these phenotypes remain to be established. Collectively, the significant genotype × treatment interactions for both AFW and primary root length indicate that the observed genotypic differences were stress-dependent under the tested conditions rather than reflecting constitutive growth differences. Moreover, the absence of significant background × genotype × treatment interactions indicates that the *rcm1t*-associated response to PEG-induced osmotic stress was statistically consistent across the three genetic backgrounds. These results provide genetic and physiological evidence that loss of *SlRCM1* function enhances osmotic-stress tolerance in tomato and support the further evaluation of *rcm1t* under field drought conditions. Nevertheless, loss of *SlRCM1* function is also associated with undesirable agronomic effects, including reduced yield and impaired fruit quality [12]. Future efforts will therefore need to determine whether the stress-tolerance benefits of *rcm1t* can be retained while minimizing these pleiotropic penalties through genetic background optimization or the combination of favorable yield- and quality-associated alleles.

In addition, *SlRCM1* is annotated as a member of the CaaX aminopeptidase family, whose members are involved in the post-prenylation processing of CaaX-containing proteins [18]. However, the proteolytic activity and specific substrates of *SlRCM1* have not yet been experimentally established. This essential post-translational modification critically regulates signal transduction, plant development, and adaptive responses to biotic and abiotic stresses by directing the subcellular targeting of client proteins, facilitating specific protein–protein interactions, and modulating the turnover and functional stability of modified substrates [18,46,47,48,49]. For example, the isoprenylated calmodulin-like protein CaM53 in *P. hybrida* requires methylation of its C-terminal isoprenylated cysteine to achieve efficient targeting and stable anchoring to the plasma membrane [20,21]. In *A. thaliana*, the dynamic balance between methylation and demethylation of isoprenylated cysteines has been shown to directly regulate the sensitivity and output strength of ABA signaling pathways [50]. Although this study did not reveal whether SlRCM1 regulates chloroplast development by modulating substrate proteins through isoprenylation, it provides some theoretical basis for further elucidating the molecular function of SlRCM1.

## 4. Materials and Methods

### 4.1. Plant Materials

The inbred lines 1624 (*SlRCM1*) and 1625 (*rcm1t*) were developed in our laboratory through successive generations of self-pollination and phenotypic selection. Line 1624 (*SlRCM1*) is a highly uniform, elite inbred line derived from over ten consecutive generations of self-pollination and rigorous phenotypic selection; line 1625 (*rcm1t*) originated as a spontaneous mutant individual identified in the progeny of 1624. Ailsa Craig (AC) and *Solanum pimpinellifolium* accession LA1589 were obtained from the Tomato Genetics Resource Center (TGRC), University of California, Davis (Davis, CA, USA). The *rcm1* loss-of-function mutant—generated via CRISPR-Cas9-mediated gene editing in the AC background (designated: AC, *rcm1*)—and the EMS-induced *rcm1* mutant in processing tomato were both kindly provided by Liu et al. [12]. The *rcm1t* mapping population was generated by crossing the inbred line 1625 (*rcm1t*) as the female parent with LA1589 as the male parent. The *rcm1t* NILs are BC6S1 lines developed by marker-assisted backcrossing. All plant materials were grown in a controlled-environment greenhouse in Haidian District, Beijing, China, across three consecutive spring–summer seasons (2022–2024).

### 4.2. Phenotypic Analysis

All field-based phenotypic assessments of tomato plants were performed using a Canon EOS 70D digital camera (Canon Inc., Tokyo, Japan). Chloroplast ultrastructure was examined by transmission electron microscopy (JEM-1230, JEOL Ltd., Tokyo, Japan) following sample preparation according to the protocol of Lu et al. [51].

### 4.3. Determination of Antioxidant Enzyme Activities and Oxidative Stress Indices

At the seedling-stage (SS) and red-ripening stages (RR), the fifth true leaf was harvested from eight individually grown tomato plants per genotype (line 1624 and line 1625), yielding eight independent biological replicates per genotype. Enzyme activity assays and all related quantitative analyses were performed in triplicate for each biological replicate. Antioxidant enzyme activities (SOD, POD, CAT) and oxidative damage markers (MDA, H_2_O_2_) were quantified spectrophotometrically using commercially available assay kits from Solarbio (Solarbio Inc., Beijing, China). The corresponding kits are as follows: SOD Activity Assay Kit (Catalog No.: BC5160), POD Activity Assay Kit (Catalog No.: BC0090), CAT Activity Assay Kit (Catalog No.: BC0200), MDA Content Assay Kit (Catalog No.: BC0020), and H_2_O_2_ Content Assay Kit (Catalog No.: BC3590). ROS levels were quantified spectrophotometrically using the Yeasen ROS Assay Kit (Catalog No.: 50101ES01; Yeasen Bio Inc., Shanghai, China).

### 4.4. Transmission Electron Microscopy Analysis

Tomato leaves—the fifth true leaf from plants at the seedling stage and the fifth true leaf from plants at the red-ripe stage—were separately collected for sample preparation, with three independent biological replicates per stage. Tomato leaf samples were immediately fixed in 2.5% (*v*/*v*) glutaraldehyde and subsequently washed with 0.1 M phosphate buffer. The samples were dehydrated through a graded ethanol series, transferred through propylene oxide, and infiltrated and embedded in Spurr resin. Polymerization was performed at 70 °C. Ultrathin sections (approximately 70 nm thick) were prepared using a Leica Ultracut R ultramicrotome (Leica Microsystems, Wetzlar, Germany). After staining, the sections were examined and photographed using a JEM-1230 transmission electron microscope (JEOL Ltd., Tokyo, Japan). Chloroplast morphology and thylakoid membrane organization were examined at different developmental stages to assess changes in chloroplast development and degradation.

### 4.5. Molecular Marker Development and Genotyping

To identify InDel and SNP variants, chromosome 8 sequences of the tomato inbred line 1625 (*rcm1t*) and LA1589 were compared following the bioinformatic pipeline established by Wei et al. [1]. PCR primers were designed to flank these variants to enable robust genotyping across parental and segregating populations. The *SlRCM1* genomic region—including both the wild-type *SlRCM1* allele and the mutant *rcm1t* allele—was amplified using the locus-specific primers and protocol described by Liu et al. [12]. Sanger sequencing was performed at Sangon Biotech (Shanghai) Co., Ltd. (Shanghai, China). To confirm the presence and precise insertion site of the LTR retrotransposon in *rcm1t*, two independent PCR assays were conducted: one targeting the 5′ junction (using primer LTR-F paired with a gene-specific reverse primer) and another targeting the 3′ junction (using primer LTR paired with a gene-specific forward primer); amplicons from both reactions were co-electrophoresed on the same agarose gel for simultaneous size verification.

### 4.6. Genetic Analysis and Fine-Mapping

Genetic analysis and preliminary mapping were conducted using 851 F_2_ individuals. For fine mapping of the *rcm1t* locus, 48 recessive homozygous mutant plants were selected from the F_2_ population. Segregation at the *rcm1t* locus was tested for goodness-of-fit to the expected 3:1 (wild-type: mutant) ratio using a chi-square test (α = 0.05) based on phenotypic scoring of all 851 F_2_ individuals. In preliminary mapping, six polymorphic InDel markers flanking the candidate region were used to genotype the 48 recessive homozygous mutants. For fine mapping, recombinants were identified by screening the entire F_2_ population with the tightly linked markers Q1 and J5; 48 recombinants were recovered—each homozygous for the *rcm1t* allele and heterozygous or homozygous only for either Q1 or J5. These recombinants were then genotyped with additional InDel and SNP markers to narrow the critical interval. Recombination rate (*r*) = [2 × (number of homozygous dominant individuals) + number of heterozygous individuals]/(2 × total number of F_2_ individuals exhibiting the recessive phenotype).

### 4.7. Gene Prediction

The candidate genes within the fine-mapped interval harboring the *rcm1t* locus were identified by querying the tomato genome annotation (ITAG 4.0) via the Sol Genomics Network (SGN; https://solgenomics.net, accessed on 15 July 2021). Subsequently, RNA was extracted following the method of Pan et al., and reverse transcription was performed, followed by PCR amplification of the coding sequences (CDS) of candidate genes within the region [52]. All Sanger sequencing was carried out by Sangon Biotech Co., Ltd. (Shanghai, China).

### 4.8. Transgenic Functional Complementation Assay

The 3500-bp native promoter of *SlRCM1*, together with the *SlRCM1* coding sequence lacking the stop codon, was cloned into the binary vector pHaoNM between the BamHI and EcoRI restriction sites to generate an SlRCM1-HA fusion construct. Tomato genetic transformation was carried out following the *Agrobacterium tumefaciens*-mediated leaf disc protocol described by Pan et al., and transgenic lines were validated by quantitative reverse transcription PCR to quantify *SlRCM1* transcript abundance [52]. Western blot analysis of the SlRCM1-HA fusion protein was performed using HA monoclonal antibody (Sigma-Aldrich, St. Louis, MO, USA; H6908), according to the protocol described by Wang et al. [15].

### 4.9. RNA-Seq Analysis

Six RNA-Seq libraries were prepared from the fifth true leaf of AC (*SlRCM1*) and AC (*rcm1*) plants at the red-ripe stage—three biological replicates per genotype (a, b, c). Sequencing was performed on the Illumina NovaSeq 6000 platform at LC-Bio Technology Co., Ltd. (Hangzhou, China). High-quality reads were aligned to the tomato reference genome (ITAG4.0), and transcript abundances were quantified using HISAT2 v2.1.0 and StringTie v2.0.6 [53,54]. Differential expression analysis was conducted using DESeq2 in R v3.0.3, with statistical significance assessed via MA-plot-guided dispersion estimation and Wald testing. Adjusted *p*-values were computed using the Benjamini–Hochberg procedure to control the false discovery rate (FDR) [55]. Fold changes were estimated from normalized DESeq2 counts—not FPKM—as FPKM is not recommended for cross-sample comparison in DEG analysis due to its lack of variance stabilization and dependence on gene length and library size. DEGs were defined as genes exhibiting |log2(fold change)| ≥ 1 and FDR-adjusted *p*-value < 0.05. Gene Ontology (GO) enrichment analysis was performed using DAVID (Database for Annotation, Visualization and Integrated Discovery; https://davidbioinformatics.nih.gov/, accessed on 9 May 2026), leveraging orthologous *Arabidopsis thaliana* annotations for functional interpretation.

### 4.10. Quantification of ABA and ABA-GE

ABA and ABA-GE were quantified via targeted ultra-performance liquid chromatography–multiple reaction monitoring tandem mass spectrometry. Leaf tissue was harvested from the third true leaves of lines 1624 and 1625 at the red-ripe stage. For each genotype, three independent biological replicates (individual plants) were analyzed, with each biological replicate subjected to three technical replicates. Approximately 100 mg of fresh tissue was homogenized in 1 mL ice-cold extraction solution (isopropanol/water, 80: 20, *v*/*v*) spiked with stable isotope-labeled internal standards (d6-ABA and d6-ABA-GE). Samples underwent sequential mechanical disruption (vortexing and bead-beating), ice-bath sonication (5 min), and incubation at −20 °C for 1 h to ensure complete metabolite extraction and protein/lipid precipitation. Extracts were then centrifuged at 14,000 × *g* for 15 min at 4 °C. An 800 μL aliquot of the supernatant was vacuum-dried to dryness, reconstituted in 160 μL methanol/water (50:50, *v*/*v*), centrifuged at 14,000 × *g* for 10 min, and the clarified supernatant filtered through a 0.22 μm polytetrafluoroethylene (PTFE) membrane prior to injection. Chromatographic separation was performed on an ACQUITY I-Class UPLC system (Waters Corporation, Milford, MA, USA) using an ACQUITY UPLC HSS T3 column (100 mm × 2.1 mm, 1.8 μm) with gradient elution at 0.3 mL/min; mobile phase A was 0.1% (*v*/*v*) aqueous formic acid and mobile phase B was 0.1% (*v*/*v*) formic acid in acetonitrile. Mass spectrometric detection was conducted on a QTRAP 6500+ triple quadrupole mass spectrometer (SCIEX, Framingham, MA, USA) equipped with an IonDrive Turbo V electrospray ionization source, operating in polarity-switching multiple reaction monitoring (MRM) mode. Quantification was performed using matrix-matched calibration curves constructed from serial dilutions of unlabeled standards spiked into blank plant matrix extracts containing fixed amounts of d6-labeled internal standards, enabling correction for matrix effects and instrument variability.

### 4.11. Yeast Two-Hybrid Assay

The *SlRCM1* CDS was cloned into the bait vector pBT3-STE between its two SfiI restriction sites, and the CDSs of NCED gene family members were individually cloned into the prey vector pPR3-N at its dual SfiI sites. Equal amounts of bait and prey plasmids were co-transformed into the yeast strain NMY51, and transformants were selected on synthetic dropout medium lacking leucine and tryptophan (SD-Leu/-Trp). The negative control consisted of co-transformation with pBT3-STE-SlRCM1 and the empty pPR3-N vector. After positive monoclonal yeast colonies emerged, individual colonies were picked and resuspended in sterile water to an OD_600_ of 0.1. Serial 10-fold dilutions (1×, 10^−1^, 10^−2^, and 10^−3^) were prepared, and 3 µL of each dilution was spotted onto SD-Leu/-Trp (selection for plasmid maintenance) and SD/-Ade/-His/-Leu/-Trp (interaction-selective) plates. Plates were incubated at 30 °C for 3–5 days, and growth was scored visually as a qualitative indicator of protein–protein interaction. All experimental and control groups were subjected to three independent biological replicates.

### 4.12. Luciferase Complementation Imaging Assay

The full-length coding sequence of *SlRCM1* or *NCEDs* was inserted into the pCAMBIA1300-nLUC or pCAMBIA1300-cLUC vector. The plasmids were verified by sequencing and used to transform *A. tumefaciens* EHA105. For co-expression assays, equal volumes of freshly cultured *A. tumefaciens* strains harboring *SlRCM1*–nLUC and *NCED2*–cLUC were mixed, adjusted to an OD_600_ of 0.8 with infiltration buffer (10 mM MgCl_2_, 10 mM MES, pH 5.6, 150 µM acetosyringone), and infiltrated into the abaxial surfaces of fully expanded leaves of 4–5-week-old *N. benthamiana* plants. For each pair of constructs, *A. tumefaciens* suspensions were infiltrated into three fully expanded true leaves (the third to fifth leaves) of each of three independent *N. benthamiana* plants. The three leaves from the same plant were considered subsamples, and each plant constituted one independent biological replicate (*n* = 3). Luminescence signals were detected using the Tanon 5200 Multi Chemiluminescent Imaging System (Tanon Science & Technology, Shanghai, China) under identical exposure settings for experimental and control samples. The assay was evaluated qualitatively.

### 4.13. Bimolecular Fluorescence Complementation Assay

The CDSs of *SlRCM1* and *NCED2* were amplified by PCR and cloned into the pEarleyGate 201-YN or pEarleyGate 202-YC, following PacI and SpeI restriction digestion [56]. Recombinant plasmids were introduced into *A. tumefaciens* strain EHA105. For co-expression assays, equal volumes of freshly cultured *A. tumefaciens* strains harboring SlRCM1–YN and NCED2–YC (or reciprocal combinations) were mixed, adjusted to an OD_600_ of 0.8 with infiltration buffer (10 mM MgCl_2_, 10 mM MES, pH 5.6, 150 µM acetosyringone), and infiltrated into the abaxial surfaces of fully expanded leaves of 4–5-week-old *N. benthamiana* plants. For each pair of constructs, *A. tumefaciens* suspensions were infiltrated into three fully expanded true leaves (the third to fifth leaves) of each of three independent *N. benthamiana* plants. The three leaves from the same plant were considered subsamples, and each plant constituted one independent biological replicate (*n* = 3). YFP fluorescence was visualized 48 h post-infiltration using a Leica DM6 CS confocal laser scanning microscope (Leica Microsystems, Wetzlar, Germany). Images were acquired with a 514 nm excitation laser, emission collected through a 525–570 nm bandpass filter, laser intensity set to 6, and photomultiplier gain fixed at 500; all settings were kept identical across samples to ensure quantitative comparability.

### 4.14. Co-Immunoprecipitation Assay

The plasmids Pro35S:*NCED2*-YFP-HA and Pro35S:*SlRCM1*-RFP-FLAG were individually transformed into *A. tumefaciens* strain EHA105. Equal volumes of exponentially grown cultures (OD_600_ = 0.8) were mixed with P19-expressing *A. tumefaciens* in infiltration buffer (10 mM MgCl_2_, 10 mM MES, pH 5.6, 150 µM acetosyringone), and the mixture was infiltrated into the abaxial epidermis of fully expanded leaves of 4-week-old *N. benthamiana* plants [57]. For each pair of constructs, *A. tumefaciens* suspensions were infiltrated into three fully expanded true leaves (the third to fifth leaves) of each of three independent *N. benthamiana* plants. The three leaves from the same plant were considered subsamples, and each plant constituted one independent biological replicate (*n* = 3). Leaf tissues were harvested 48 h post-infiltration. Total proteins were extracted in IP lysis buffer (50 mM Tris–HCl, pH 7.5; 150 mM NaCl; 0.1% (*v*/*v*) CA-630; 20% (*v*/*v*) glycerol; 5 mM DTT; and one tablet of protease inhibitor cocktail (Roche Diagnostics GmbH, Mannheim, Germany; 04693132001) per 50 mL buffer), followed by centrifugation at 16,000 × *g* for 15 min at 4 °C to remove debris. Immunoprecipitation was performed using anti-FLAG magnetic beads (Lablead, Beijing, China; FNM-25-1000), with overnight incubation at 4 °C under gentle rotation. Beads were washed three times with IP wash buffer (50 mM Tris–HCl, pH 7.5; 150 mM NaCl; 0.1% (*v*/*v*) CA-630), and bound protein complexes were eluted with 2× SDS loading buffer. Eluates were resolved by 10% SDS–PAGE and transferred onto polyvinylidene fluoride (PVDF) membranes (Immobilon-P, Merck Millipore, Burlington, MA, USA; IPVH00010). Immunoblotting was carried out with anti-HA antibody (Sigma-Aldrich, St. Louis, MO, USA; H6908; 1:2000) or anti-FLAG antibody (Lablead, Beijing, China; F1005; 1:2000), and chemiluminescent signals were detected using Clarity Max Western ECL Substrate (Bio-Rad Laboratories, Hercules, CA, USA; 1705062) and visualized on a Tanon 5200 Multi Chemiluminescent Imaging System (Tanon Science & Technology, Shanghai, China).

### 4.15. Osmotic Stress Treatment

Seed germination assays were performed in sterile Petri dishes: surface-sterilized seeds were uniformly distributed on double-layered filter paper saturated with distilled water or PEG-6000 solution, and incubated under constant dark conditions at 26 °C. The solution was refreshed every 24 h, and germination was scored daily as radicle emergence ≥ 2 mm beyond the seed coat; data were collected over five consecutive days. The seedling-stage osmotic stress assay was performed in a controlled greenhouse: tomato plants at the three-true-leaf stage were subjected to tray-soaking treatment with either distilled water or 10% PEG-6000 solution, repeated every 48 h; phenotypic traits—including fresh weight, maximum primary root length, plant height, and root dry weight—were quantified after 10 days of treatment. All the above experiments were conducted with three independent biological replicates.

### 4.16. Scanning Electron Microscopy Analysis

For each genotype and treatment, three plants were sampled, including AC (*SlRCM1*) and AC (*rcm1t*) plants under well-watered and osmotic stress conditions. The third true leaf from each plant was collected and cut into small pieces (approximately 1 mm^3^), which were immediately fixed in 2.5% glutaraldehyde at 4 °C overnight. After fixation, the samples were rinsed three times with 0.01 M phosphate-buffered saline (PBS, pH 7.4) for 15 min each. The samples were then dehydrated through a graded ethanol series (30%, 50%, 70%, and 90%) for 15 min at each concentration, followed by 100% ethanol for 20 min, and finally transferred to fresh 100% ethanol. The dehydrated samples were dried in a critical-point dryer (EM CPD300, Leica Microsystems, Wetzlar, Germany). The dried specimens were mounted on sample stubs using conductive carbon adhesive and sputter-coated with platinum for approximately 70 s using an ion sputter coater (Mini Coater, SuPro Instruments, Shanghai, China). Stomatal morphology was imaged using a scanning electron microscope (Sigma 360, ZEISS, Oberkochen, Germany).

### 4.17. Measurement of Stomatal Aperture

Stomatal aperture was quantified as stomatal pore area using Fiji (ImageJ 1.54f; National Institutes of Health, Bethesda, MD, USA) from SEM micrographs captured under identical imaging conditions, including the same developmental stage, leaf region, and magnification. The boundary of each stomatal pore was manually delineated, and the enclosed pore area was measured. Ten stomata were analyzed per biological replicate, with three independent biological replicates per treatment.

### 4.18. Data Statistical Analysis

Apart from the data in Figure 11, all other quantitative data were calculated using IBM SPSS Statistics 20.0 to obtain descriptive statistics, including means and standard deviations. A two-tailed non-paired *t*-test was also conducted to determine whether there were statistically significant differences in the means between the groups. Chi-square goodness-of-fit tests were conducted using the online platform SPSSPRO (https://www.spsspro.com, accessed on 12 April 2026), with significance thresholds set at *p* < 0.05. For the germination assay shown in Figure 11a, data were analyzed using a two-way analysis of variance (ANOVA), with genotype (*SlRCM1* and *rcm1t*) and PEG-6000 concentration as fixed factors, including the genotype × PEG concentration interaction. For the seedling osmotic-stress assays shown in Figure 11b,c, aboveground fresh weight and maximum primary root length were analyzed separately using three-way ANOVA, with genetic background (AC, 1008, and 1009), genotype (*SlRCM1* and *rcm1t*), and treatment (control and 10% PEG-6000) as fixed factors. All two-way interactions and the background × genotype × treatment interaction were included in the models. When significant interactions were detected, simple-effect comparisons were performed from the corresponding factorial model with Šídák adjustment for multiple comparisons. Data are presented as means ± SD of nine independent biological replicates. Statistical significance was defined as *p* < 0.05. All statistical analyses were performed using GraphPad Prism 9.5.1 (GraphPad Software, San Diego, CA, USA) for Windows.

## 5. Conclusions

This study identified and cloned *rcm1t*, a naturally occurring loss-of-function allele of *SlRCM1* caused by the insertion of an LTR retrotransposon, which causes premature chloroplast senescence during late leaf development. Quantification of ABA and ABA-GE revealed that the loss of *SlRCM1* function was associated with a significant increase in ABA levels, accompanied by a significant decrease in ABA-GE levels. Protein–protein interaction assays demonstrated that SlRCM1 interacts with SlNCED2. Osmotic stress assays showed that the *rcm1t* mutant exhibited significantly enhanced tolerance to osmotic stress. Collectively, these findings support a working hypothesis that SlRCM1 may be involved in modulating ABA homeostasis through its interaction with SlNCED2, with loss of SlRCM1 being associated with elevated ABA accumulation and enhanced osmotic stress tolerance in tomato. However, the biochemical and causal relationships underlying these observations remain to be established.

## Figures and Tables

**Table 1:** Predicted genes in the *rcm1t* region.

Gene Name	Position on SL4.0ch08	Putative Function
*Solyc08g004000*	403–3245(+)	Unknown protein
*Solyc08g004005*	10,075–14,531(+)	Unknown protein
*Solyc08g005000*	16,719–20,439(−)	Glucan endo-1 3-beta-glucosidase 3
***Solyc08g005010***	26,915–33,223(−)	**CAAX amino terminal protease** **(*L1/SlRCM1*)**
*Solyc08g150100*	34,051–36,325(+)	microspore-specific promoter 2
*Solyc08g005020*	36,737–39,729(+)	nucleolin
*Solyc08g005030*	40,225–43,679(+)	Splicing factor 3B subunit 5
*Solyc08g005040*	44,420–44,758(−)	hypothetical protein
*Solyc08g005050*	44,785–49,982(−)	transcription factor MYC2

## Data Availability

The RNA sequencing datasets generated in this study have been deposited in the Sequence Read Archive (SRA) under the accession number PRJNA1497919.

## Supplementary Materials

The following supporting information can be downloaded at: https://www.mdpi.com/article/10.3390/ijms27188071/s1.
