# Supplementary material for: rcm1t, a Loss-of-Function Allele of SlRCM1, Causes Premature Chloroplast Senescence and Is Associated with Enhanced Osmotic Stress Tolerance in Tomato
Source: Int J Mol Sci. 2026 Sep 10;27(18):8071. doi: 10.3390/ijms27188071 (PMC13606836; doi:10.3390/ijms27188071)
Supplement: Supplementary file 1 [file ijms-27-08071-s001.zip › Figure S1-S9.pdf]

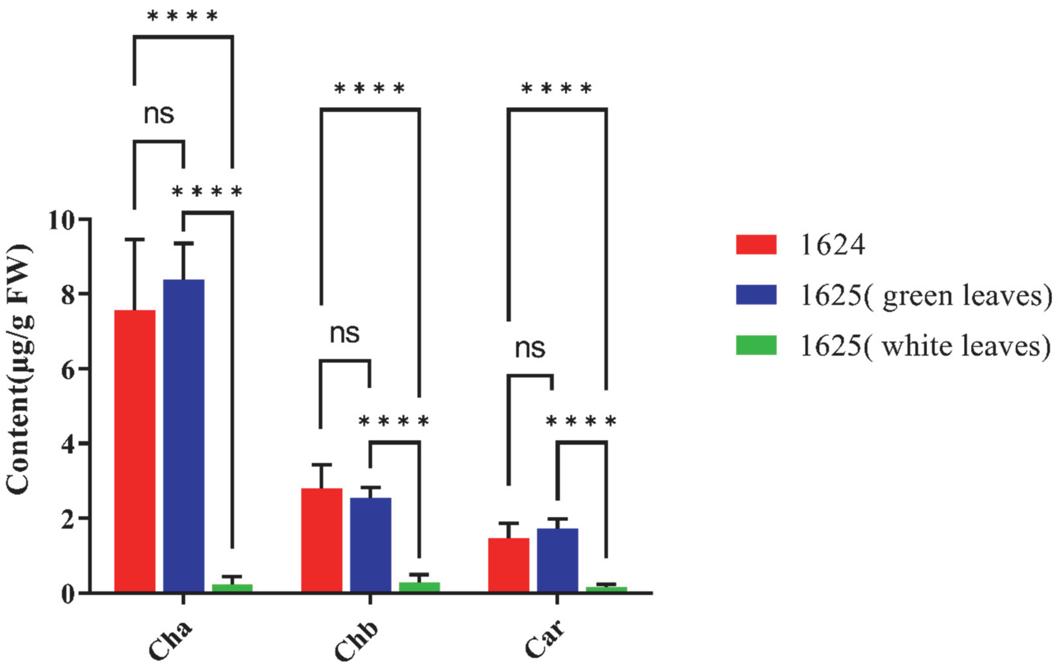


**Figure S1.** Photosynthetic pigment quantification in leaves of mutant and wild-type plants across distinct physiological stages. Asterisks indicate a significant difference (****, $p < 0.0001$).

(a)

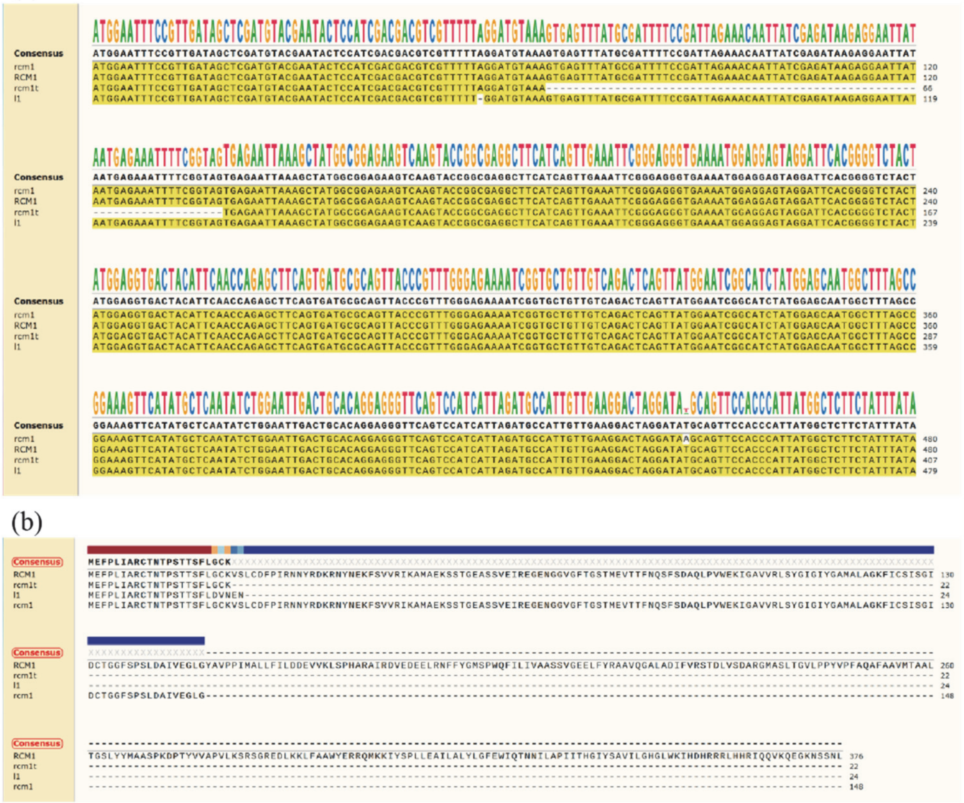


**Figure S2.** Comparative alignment of the coding DNA sequences and corresponding amino acid sequences across multiple *SlRCM1* allelic variants. (a) Multiple-sequence alignment of *SlRCM1* allelic variants coding DNA sequences. (b) Amino acid sequence alignment.

CAAACTTCTAACAGCTGAAACGACGCCGCTTGCTCATTTTCCGATTCGCTTGTCAGTTTAGACCGGAGTGTTTTGTAAATATGGAATTTCCGTTGATAGCTCGATGTACGAATACTCCATCGACGACGTCGTTTTTAGGATGTAAA

GTGAGTTTATGCGATTTTCCGATTAGAAACTGTTGAATGCCTTGAATCGGACCCGCTACAAACAGAAAGGACGGGGGTCTCGCTGCCCGGTCAGCGAGTCGGGGGGTCCAGGGGGGCGACGCGCCCCCTGGCCTGGGGGTCCG

GGGGGGCGGAGACGCCCCCGGGCCGACGGTATACAATGTTGTTGTATTGGGCCCTTAATTTTCTGTTGATTCTGTATGTTGGGCCCAAGCCTGTTAGGGCGTAGCTTAGCACTATATATAGACGCTATGGGAAACCCTATTCTGTA

ATTCTGTTTTTGCCTCTCCATAATAAAACTGCTCCCTCTCTTCCCGTGGACGTAGCCAATTTGTTGGTGAACCACGTAAATCTGTTGTCTTATTTTTCGCGTTTATATTTTCTCGTATTATCTCAAATTCCGCACAACAAATTGGTATC

AGAGCCTCTCGGTTAATCGGTGTTCTTGGAGAATTCGAGATGTCTGCTTTGAACGTGAAAATCGACAAATTCACAGGGAGGAACAGTTTCAGTTTATGGCAGATCAAGATGCGGGCCTTGTTGAAACAGCAAGGCTTCTGGGCG

CCGTTGTCGAAAGACAAGAACGCCGTCGTTACTCCTGAGATGGCGATTCTGGAGGAAAAGGCGCACTCGACGATCATGCTGTGTCTCGCGGATGACGTCATCACGGAGGTCTCGGATGAAGAGACTGCTGCTGGTCTGTGGTTG

AAGCTGGAGAGTTTGTACATGACAAAATCTCTAACCAACAAGCTGCTTCTGAAACAACGTCTATTCGGTTTACGAATGGCTGAAGGTACACAACTCAGGGAACACTTAGAGCAATTGAATACTTTGTTATTAGAATTGCGTAAT

ATCGATGTGAAGATCGAGGATGAAGATGCTGCCCTGATTCTGTTAGTATCTCTCCCAATGTCGTTTGAGAATTTTGTTCAATCGTTCATTGTTGGGAAAGATACTGTGTCACTGGAAGAAGTCAGATCAGCCCTTCATAGCAGGGA

ATTACGGCATAAGGCTAACGGCACAAGTACGGACATACAGCCTTCCGGTCTGTTCACCAGTAGCAGAAAGGGAAGGAAAAACGGCGGAAAGAAAAATAAGCCGATGTCGAAGGGTGCAAAGCCGGATGATGTTTGTAATTA

CTGCAAGGAGAAGGGACATTGGAAATTTGATTGTCCGAAGAAGAAGAAGCAATCGGAAAAACAATCAGTGTCTGCTGCTGTTGCTGAAGAAGACACCAATTCTGAAGAAGATATTGCCCTAGTTGCGGATGAGCACACTCATC

ATTCAGATGTGTGGGTTCTTGATTCTGGGGCATCCTATCACATCTGTCCTAGGAGAGAGTGGTTCACGACTTATGAGCAGGTAGACGGAGGCAGCATCTCGATGGCCAACAGTTCTGTCTGCAAGGTGGTTGGGACAGGCTCGAT

CAAGATAAGGACACATGACGGTAGCTTCTGCACATTGAACGAGGTCAGGCACGTTCCATTGATGACGAAAAATCTGATATCTCTCAGTCTTTTGGACAGCAAGGGATTCAGCTGGTCGGGAAAAGATGGAGTCTTGCGGGTCTG

GAAGGGTTCAAATTTGATTCTGAAAGGTGTCATGCGTGGTACTTTGTATTTTCTACAAGGTTCCACGGTTACAGGTTCAGCCCATGTTGCATCGTCAGAATTTCACCAGAAGGATATGACTAAGTTATGGCACATAAGACTTGGTC

ATATGGGTGAAAGAGGGATGCAAATTCTGTCAAAGGAGGATTTACTTGCTGGTCATAAGGTTAAGAGCCTAGAGTTTTGTGAACATTGTGTTTTTGGAAAACTACATCGCAACAAGTTTCCAAAGGCCATTCATAGAACAAAAG

GCACACTTGATTATATCCATTCTGATTGCTGGGGTCCATGCCGTGTTGAGTCTTTGGGAGGCTGCAGATTTTTTGTGTCCATTATTGATGACTACTCAAGGATGACTTGGGTGTACATGATGAAGCATAAAAGTGAAGCCTTCCAG

AAGTTCAAGGAGTGGAAAATTTTGATGGAAAATCAAACAGGGAAGAAGATCAAGAGGTTGCGAACTGATAATGGGCTGGAATTCTGTTGGTCTGAATTTGATCAATTCTGTAAGGATGAAGGGATTGCTCGACATCGCACAGT

CAGAAATACACCACAGCAGAACGGTGTAGCTGAGCGGATGAATCAAACACTTCTGGAGAGAGCAAGGTGCATGCTCTCTAATGCTGGGCTAGATAGAAGATTCTGGGCAGAAGCGGTTAGTACAGCTTGCTACTTGATTAACC

GCGGACCACATACAGGTATACAGTGCAAGACACCTATGGAGATGTGGTCTGGAAAAGCTGCTGATTATTCAAATCTGAAAGCTTTTGGTTGTACGGCTTACTATCACGTCAGTGAAGGTAAGTTAGAACCAAGAGCTAAAAAG

GGAGTATTTGTGGGCTACGGAGATGGAGTGAAAGGTTTCAGAATCTGGTCTCCAGCAGAAAAGAGGGTTATTATGAGCAGGAACGTTGTCTTTGATGAAAGTCCTCTGCTTAGAACCATTGTGAAGCCTACAACTACGTCAGAA

ACTGGGAGTCTTGACAAACAGGTGGAGTTTCAAGTCATTCAGAACGAGAGCGATTTAAAGGAACCTGAAGAGGAGGATCAAGAGCCACAGACTGAAACTGATATTCCAGAATCTATGCCATCAGATATCCATCAGAGTATAG

CTCAAGATCGGCCAAGGAGGGTTGGAGTTCGGCCACCTACGAGGTATGGTTTTGAGGACATGGTGGGTTATGCACTGCAGGTTGCTGAAGAGGTAGATACATCTGAGCCGTCTACTTACAAAGAAGCCATTTTAAGTTCTGATT

CTGAAAAATGGTTTGCCGCTATGGGAGATGAGATGGAGTCCCTACACAAGAATCAGACATGGGATCTGGTCATACAGCCTTCGGGGAGAAAGATTATTACTTGCAAATGGGTTTTCAAGAAGAAGGAAGGGATATCACCAGCA

GAAGGAGTCAAGTATAAAGCCAGGGTTGTTGCCAGAGGTTTCAACCAAAGAGAGGGAGTGGACTATAATGAGATCTTCTCACCAGTGGTCAGACATACTTCCATCCGAGTGTTACTAGCGATAGTTGCACATCAGAATCTGGA

GCTTGAACAACTTGATGTGAAGACAGCGTTTCTACATGGAGAGTTGGAGGAAGAGATATACATGACTCAGCCGGATGGTTTCCAAGTTCCAGGGAAGGAAAATCACGTCTGCAAGTTGAAGAAGTCCTTATATGGACTTAAGC

AGTCTCCAAGGCAGTGGTATAAAAGGTTTGACAGCTATATGGTGAAGTTGGGCTATACTCGGAGCTCATATGATTGTTGTGTCTACTACAATAGGCTCAATGATGATTCATTCATCTATCTGGTGCTTTATGTAGATGATATGTTG

ATAGCTGCAAAGAAGAAGTATGACATTCAGAAGCTGAAGGGTTTACTTAGTGCTGAGTTTGAGATGAAGGATCTGGGAGCCGCTCGGAAGATTTTAGGGATGGAGATCATTAGAGACAGAGAGAGAAGGAAACTTTTCTTGTC

ACAGAGAAGCTACATTCAGAAGGTCTTGGCGAGGTTTGGCATGTCTTCATCTAAGCCCATTGATACCCCCAGTGCTGCCAATATCCATCTCACTGCCATGTTCGCTCCACAGTCAGAAGAAGAGAAGGAGTATATGTCACGAGT

CCCTTATGCCAGTGCCGTAGGAAGTTTGATGTATGCTATGGTCTGTACAAGGCCAGATTTAGCACATGCAGTCAGTGTAGTGAGCAGATTCATGGGACAACCAGGGAGAGAACATTGGCAGGCTGTGAAGAGAATTTTCCGGTA

CCTTAGAGGTACATCTGACGTTGGTCTCATTTATGGAGGTGATACTCAGTGCTTGGTTACTGGCTATTCTGATTCAGACTATGCTGGAGATGTTGACACAAGAAGATCGATGACTGGCTATGTGTTTACCCTTGGAGGATCTGTCG

TCAGTTGGAAGGCAACTTTGCAACCTACAGTGACTTTGTCTACTACGGAAGCGGAGTACATGGCCTTGACAGAGGCTGCAAAAGAAGGGATTTGGTTGAAAGGGCTGGTTAGTGATCTTGGTCTGCATCATGATCAGGCTACGG

TGTATTGTGACAGTTTGAGCGCAATTTGTCTAGCCAAGGATCAAGTCCATCATGAGAGAACCAAGCATATTGACGTAAGGTATCATTTTCTAAGAAGTGAGAAGAGAATCAAGGTGAAGAAAGTAGGAACTGCTGATAATCCT

GCTGATATGTTCACAAAGCCGGTTCCACAGAGCAAGTTTCAACACTGTTTGGACTTGCTCAACATCAGAAGCTGTTAATTGCCCTGCGGGGCAACTCTGAGGAAGAGGGGGAGGCTTGGCACTATCATAGTGCGTCTGAAGAAT

CTGTTCGGAGAATTCAAGTCAAGGTGGAGATTTGTTGAATGCCTTGAATCGGACCCGCTACAAACAGAAAGGACGGGGGTCTCGCTGCCCGGTCAGCGAGTCGGGGGGTCCAGGGGGGCGACGCGCCCCCTGGCCTGGGGGTC

CGGGGGGGCGGAGACGCCCCCGGGCCGACGGTATACAATGTTGTTGTATTGGGCCCTTAATTTTCTGTTGATTCTGTATGTTGGGCCCAAGCCTGTTAGGGCGTAGCTTAGCACTATATATAGACGCTATGGGAAACCCTATTCTG

TAATTCTGTTTTTGCCTCTCCATAATAAAACTGCTCCCTCTCTTCCCGTGGACGTAGCCAATTTGTTGGTGAACCACGTAAATCTGTTGTCTTATTTTTCGCGTTTATATTTTCTCGTATTATCTCAAATTCCGCACAACAGAAACAAT

TATCGAGATAAGAGGAATTATAATGAGAAATTTTCGGTAGTGAGAATTAAAGCTATGGCGGAGAAGTCAAGTACCGGCGAGGCTTCATCAGTTGAAATTCGGGAGGGTGAAAATGGAGGAGTAGGATTCACGGGGTCTACTAT

GGAGGTGACTACATTCAACCAGAGCTTCAGTGATGCGCAGTTACCCGTTTGGG

**Figure S3.** Sequence of the long terminal repeat retrotransposon. Green letters represent the genome sequence of the *SlRCM1* gene, whereas gray letters represent the direct repeat sequence of the retrotransposon. Red letters represent the target site duplication sequence.

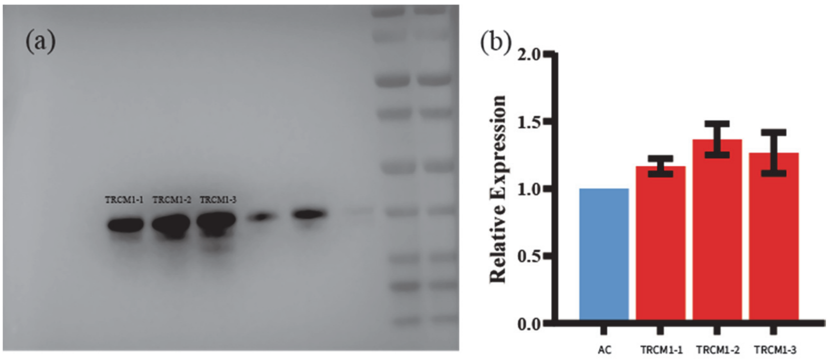


**Figure S4.** Molecular confirmation of *SlRCM1* transgenic lines. (a) Western blot analysis results. (b) qRT-PCR quantification results. Three independent transgenic lines were designated TRCM1-1, TRCM1-2, and TRCM1-3.

.

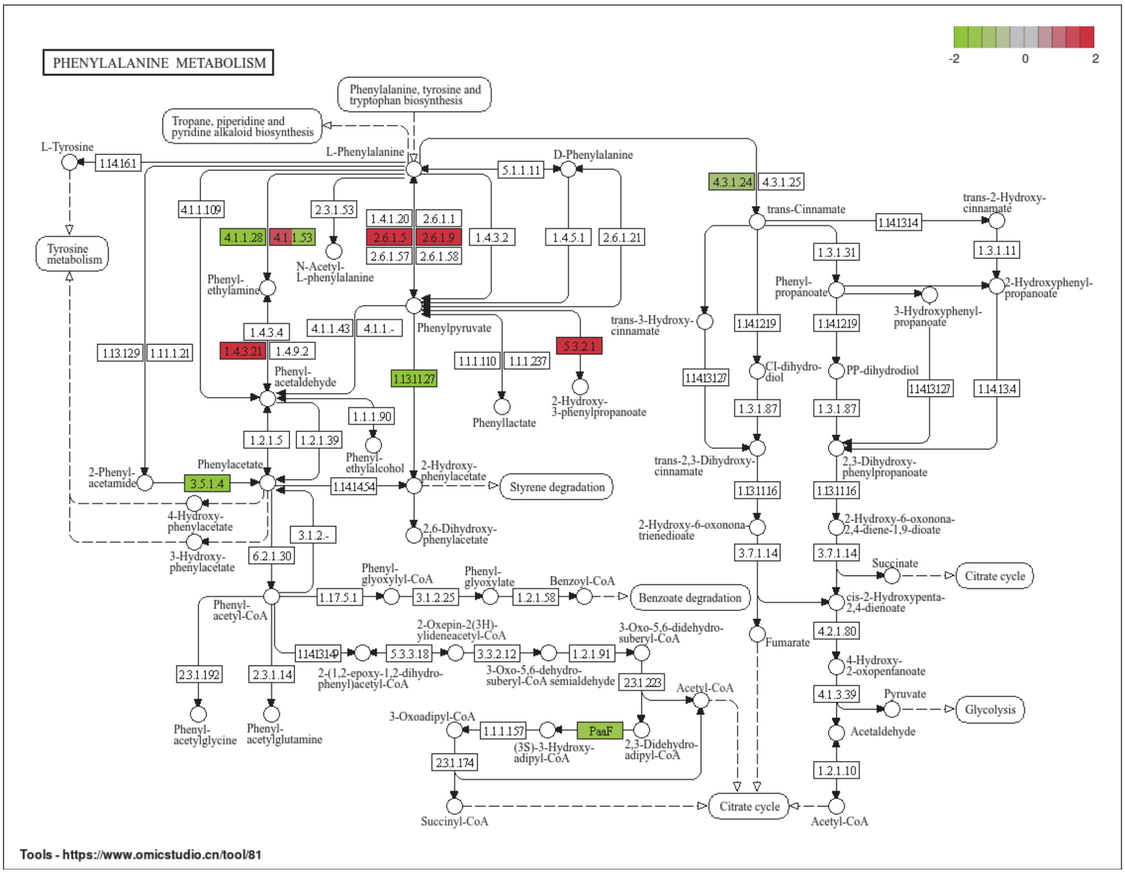


**Figure S5.** DEGs of ko00360.

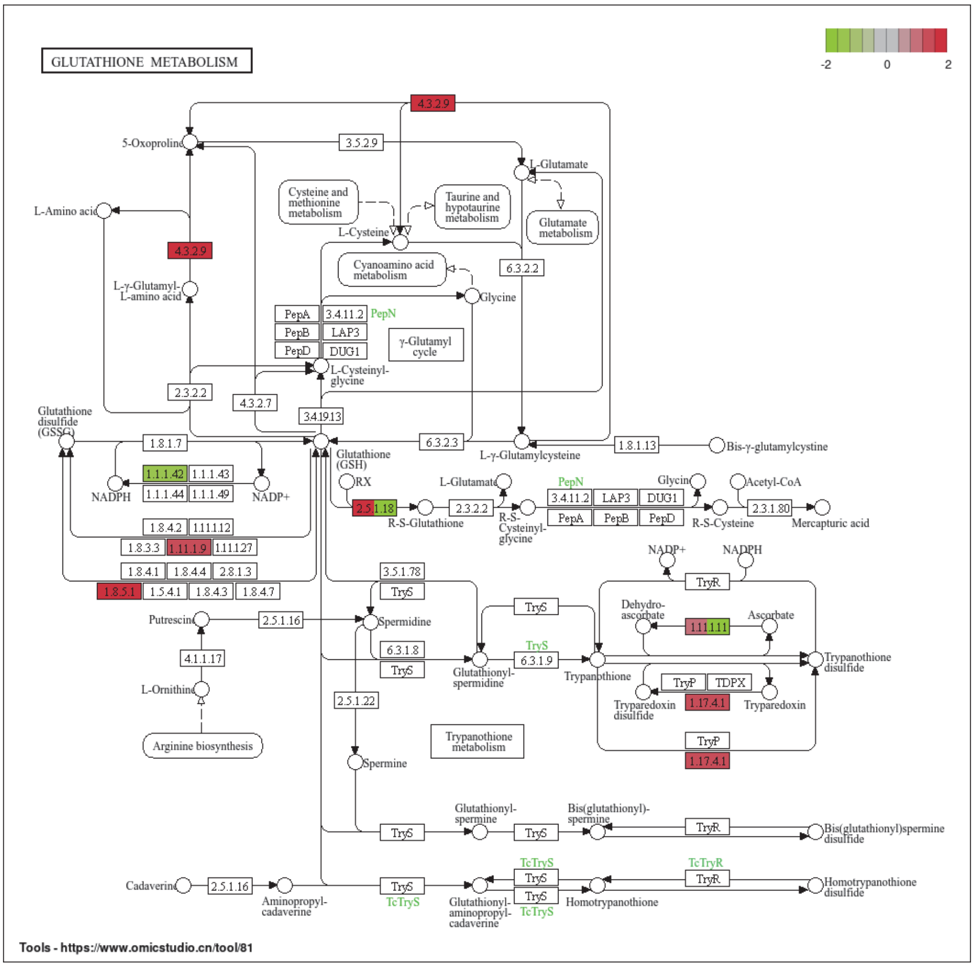


**Figure S6.** DEGs of ko00480.

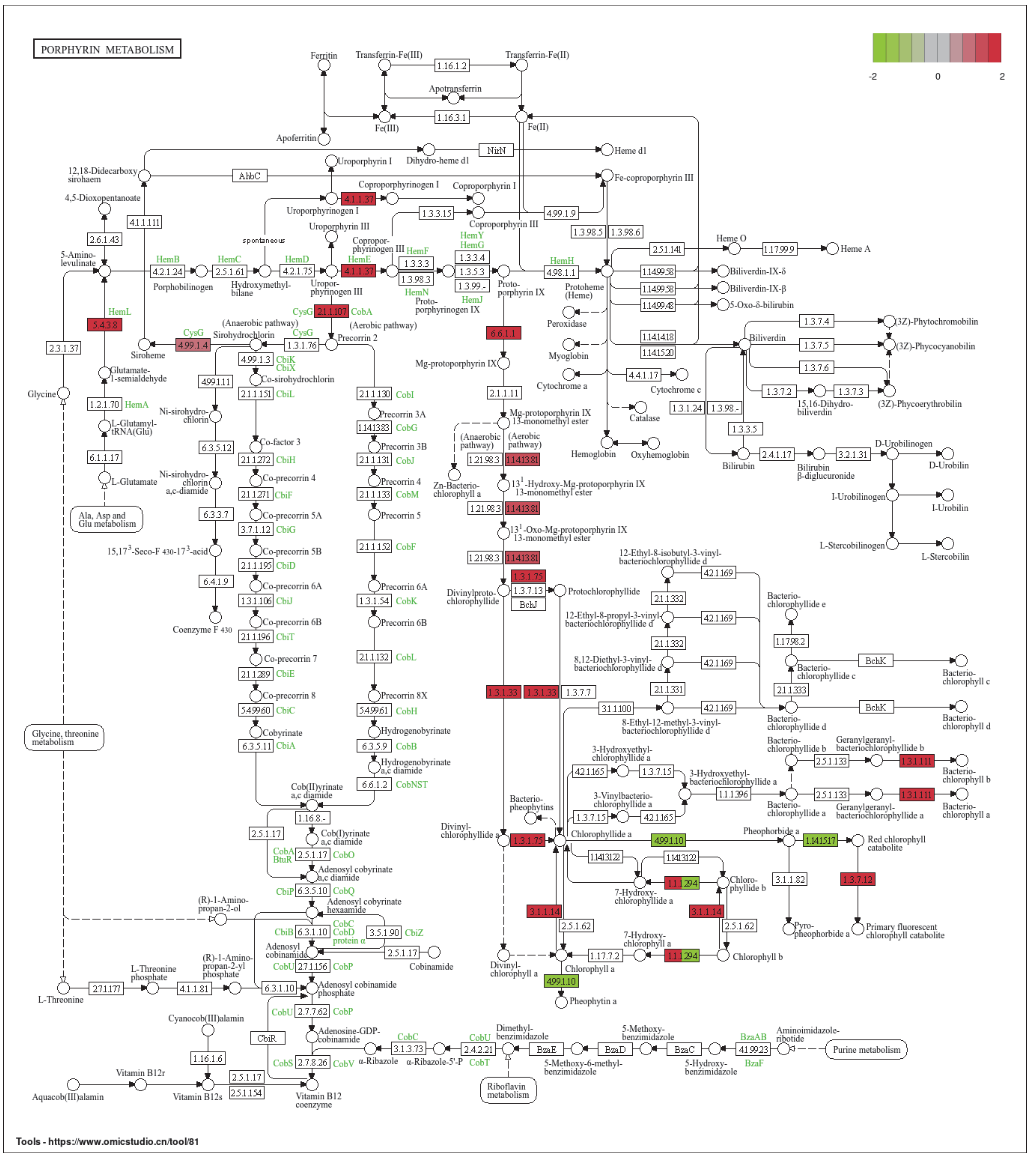


**Figure S7.** DEGs of ko00860.

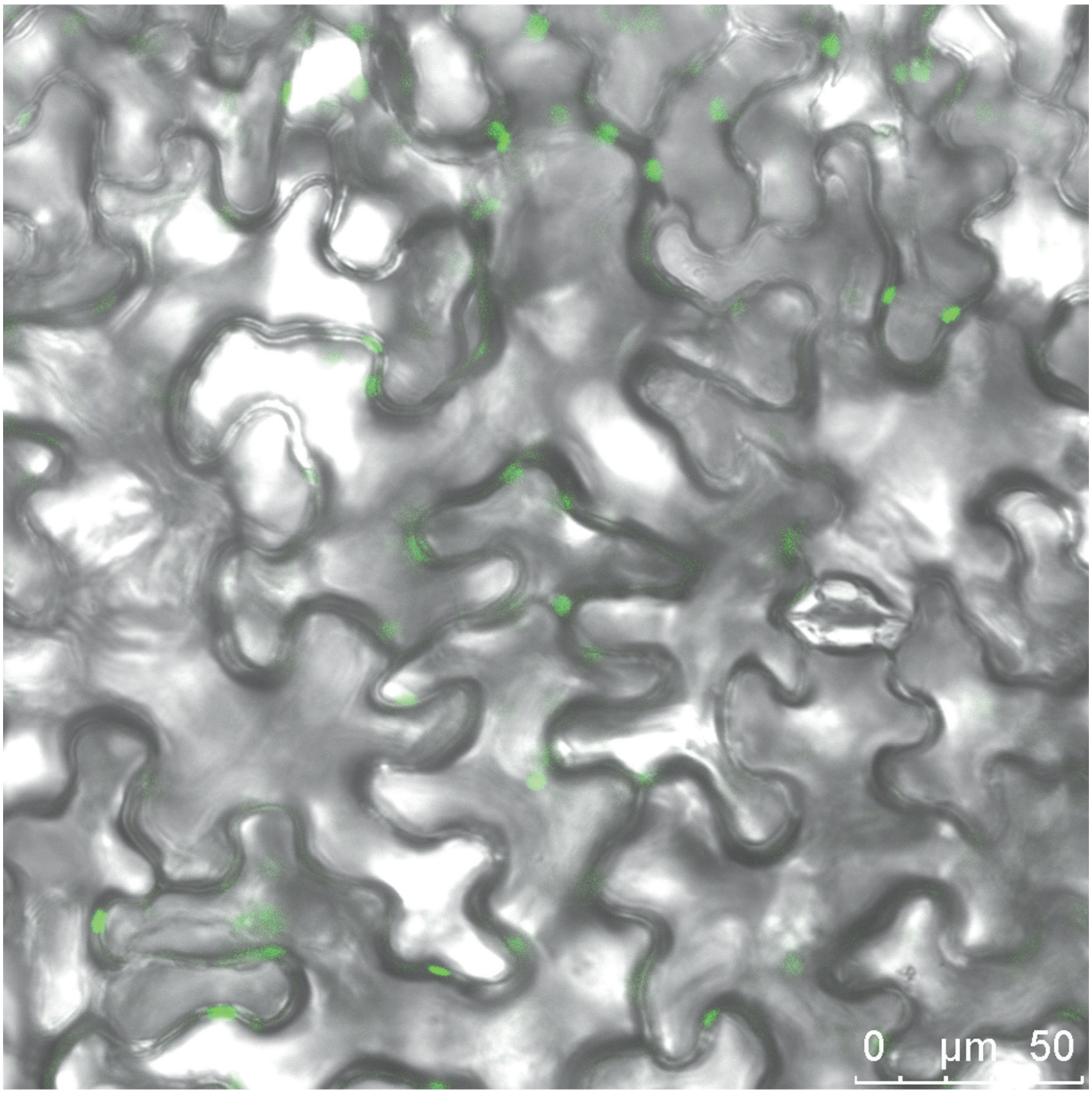


**Figure S8.** SlNCED2 localizes to the chloroplast.

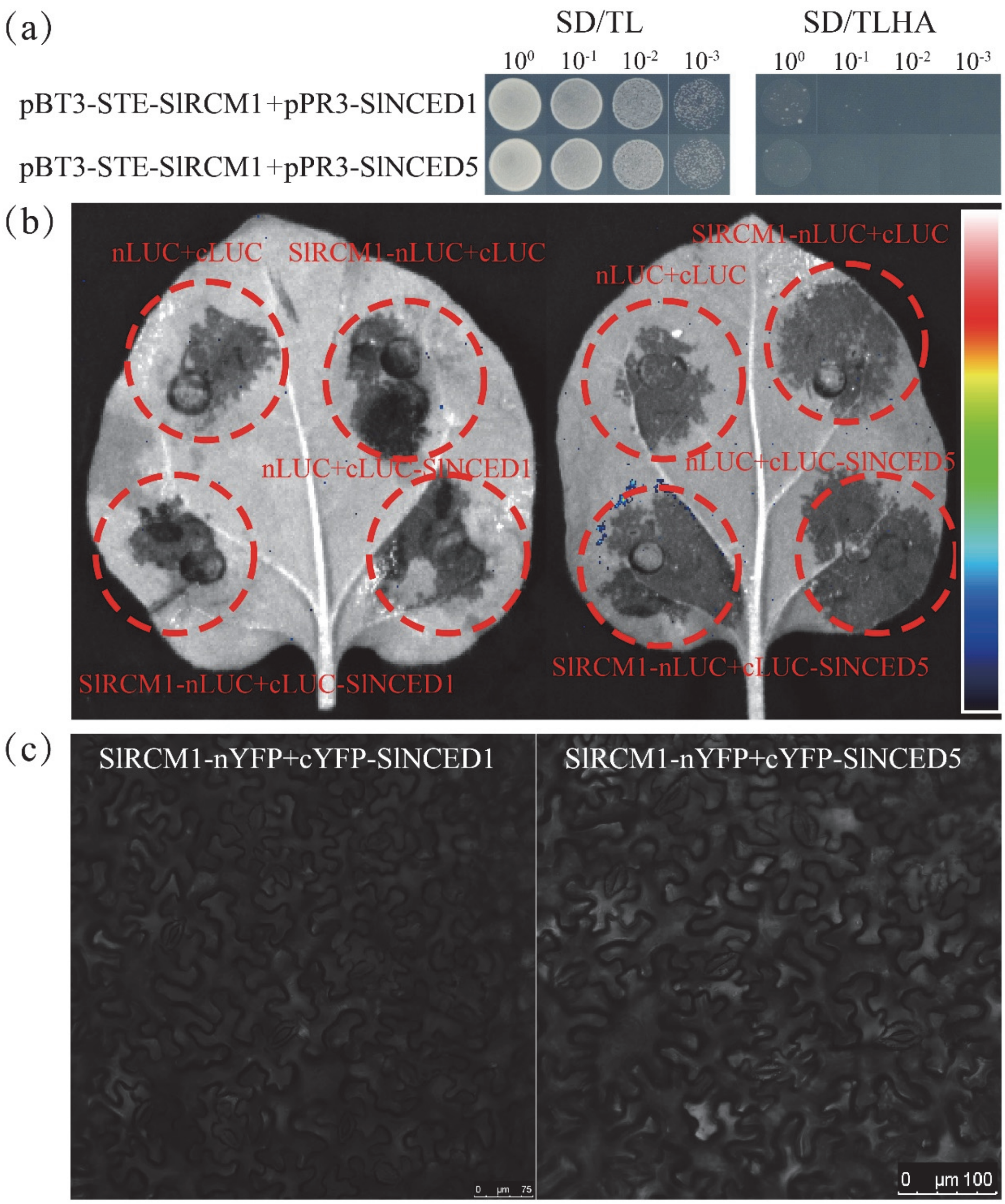


**Figure S9.** Negative controls and absence of interaction in protein–protein interaction assays. (a) Y2H assay testing the interaction between SlRCM1 and SlNCED1 or SlNCED5. (b) LCI assay testing the interaction between SlRCM1 and SlNCED1 or SlNCED5. (c) Negative controls in BiFC assays.
